# Design, synthesis and bioactivity study on oxygen-heterocyclic-based pyran analogues as effective *P*-glycoprotein-mediated multidrug resistance in MCF-7/ADR cell

**DOI:** 10.1038/s41598-024-56197-w

**Published:** 2024-03-31

**Authors:** Ashraf H. F. Abd El-Wahab, Rita M. A. Borik, Al-Anood M. Al-Dies, Ahmed M. Fouda, Hany M. Mohamed, Raafat A. El-Eisawy, Ahmed Mora, Mohammed A. A. El-Nassag, Ahmed M. Abd elhady, Ahmed A. Elhenawy, Ahmed M. El-Agrody

**Affiliations:** 1 Chemistry Department, Faculty of Science, Jazan University, B.O. Box 2097, Jazan, 45142 Kingdom of Saudi Arabia; 2 Chemistry Department, Umm Al-Qura University, Al-Qunfudah University College, 21912 Al-Qunfudah, Saudi Arabia; 3 Chemistry Department, Faculty of Science, King Khalid University, 61413 Abha, Saudi Arabia; 4 Chemistry Department, Faculty of Science, Al-Azhar University, Nasr City, 11884 Cairo Egypt; 5 Chemistry Department, Faculty of Science, Al-Baha University, 65528 Al-Baha, Saudi Arabia; 6 Chemistry Department, Faculty of Science, Al-Baha University, 65528 Al-Bahah, Saudi Arabia

**Keywords:** Biochemistry, Drug discovery, Biochemistry, Medicinal chemistry, Organic chemistry

## Abstract

*P*-glycoprotein (*P*-gp) imparts multi-drug resistance (MDR) on the cancers cell and malignant tumor clinical therapeutics. We report a class of newly designed and synthesized oxygen-heterocyclic-based pyran analogues **(4a–l)** bearing different aryl/hetaryl-substituted at the 1-postion were synthesized, aiming to impede the *P*-gp function. These compounds (**4a–l**) have been tested against cancerous PC-3, SKOV-3, HeLa, and MCF-7/ADR cell lines as well as non-cancerous HFL-1 and WI-38 cell lines to determine their anti-proliferative potency.The findings demonstrated the superior potency of **4a–c** with 4-F, 2-Cl, and 3-Cl derivatives and **4h,g** with 4-NO_2_, 4-MeO derivatives against PC-3, SKOV-3, HeLa, and MCF-7/ADR cell lines.Compounds **4a–c** were tested for *P*-gp inhibition and demonstrated significant vigour against MCF-7/ADR cells with IC_50_ = 5.0–10.7 μM. The Rho123 accumulation assay showed that compounds **4a–c** adequately inhibited *P*-gp function, as predicted. Furthermore, **4a** or **4b** administration resulted in MCF-7/ADR cell accumulation in the S phase, while compound **4c** induced apoptosis by causing cell cycle arrest at G2/M. The molecular docking was applied to understand the likely modes of action and guide us in the rational design of more potent analogs. The investigate derivatives showed their good binding potential for *p*-gp active site with excellent docking scores and interactions. Finally, the majority of investigated derivatives **4a–c** derivatives showed high oral bioavailability, but they did not cross the blood–brain barrier. These results suggest that they have favorable pharmacokinetic properties. Therefore, these compounds could serve as leads for designing more potent and stable drugs in the future.

## Introduction

**Figure 1:**
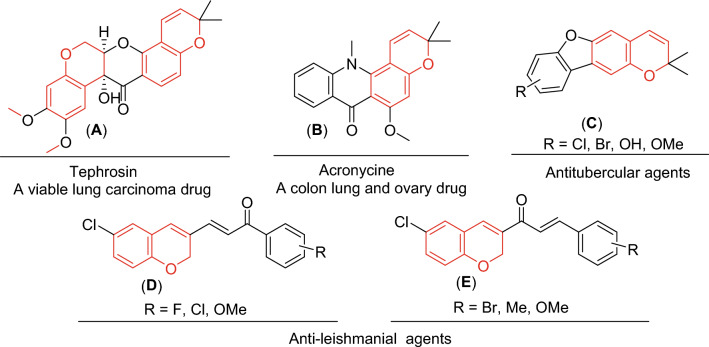
Chromene-based compounds (red highlighted) with biological activities.

Over the last few decades, there has been a significant increase in the incidence of cancer, making it one of the leading causes of death in developing nations. Cancer’s characterization incorporates the disorderly progression of abnormal cells, triggering apoptosis (a critical condition that grants tumor development) and metastasis (the formation of malignant growths away from the pioneering site)^1^. Several strategies have been employed to tackle the impacts of this disease. These approaches to cancer therapeutics encompass surgery, biological therapy, and radiotherapy^2^. Additionally, one of the most renowned therapeutics is chemotherapy, which applies to a variety of protocols^3,4^. However, this methodology’s efficiency fluctuates per patient, mistakenly targets cells exhibiting normalcy, and reveals severe side effects, including extreme fatigue, hair loss, vomiting, gastrointestinal diseases, immune suppression, and kidney/liver disease and destruction. These obstacles have driven the discovery of sufficient antitumor agents. Numberless fused chromene compounds were used for the treatment of several cancers diseases. For example, Tephrosin (**A**), a viable lung carcinoma drug, Acronycine (**B**), a colon lung, and ovary drug, which subsequently causes apoptosis through microtubule polymerization^5^. In addition, chromene derivatives (**C**) also proved to be promising antitubercular agents, especially the compound bearing a methoxy group in 9-position^6^. Chlorochromene derivatives (**D**) and (**E**) showed anti-leishmanial activity. They were nontoxic and promising leads for these protozoan infections^7^ as illustrate in (Fig. 1).

**Figure 2:**
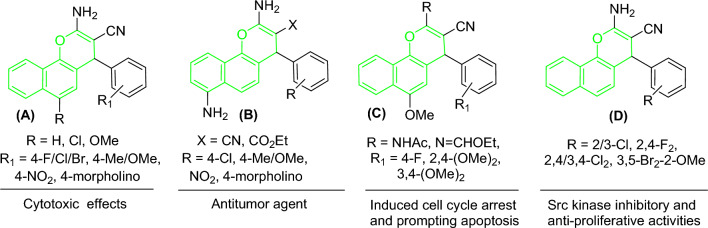
Structure of some bioactive 4*H*-benzo[*h*]chromene derivatives (green highlighted).

Among the candidates for this objective, 4*H*-benzo[*h*]chromenes scaffolds (Fig. 2) have been reported as promising candidates to develop anticancer drugs. These include, *β*-enaminonitrile and its 6-Cl/OMe (**A**)^8–10^, 3-carbonitrile/carboethoxy substituents (**B**) which act as antitumor agents^8^, 2-NHCOCH_3_/N=CHOEt-6-OMe derivative (**C**) induced cell cycle arrest^9,11,12^ and halogen derivatives of 4*H*-benzo[*h*]chromene (**D**), leading to potent anticancer analogs that targeting the *c*-Src Kinase enzyme^13^.

**Figure 3:**
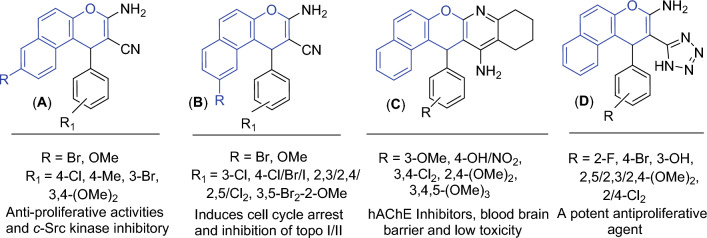
Structure of some 1*H*-benzo[*f*]chromene derivatives (blue highlighted) with cytotoxic and apoptotic effects.

In addition, 1*H*-benzo[*f*]chromene is one of the most supportive heterocyclic systems with a range of pharmaceutical operations. 8-Br/OMe substituents (**A**) resist *c*-Src kinase, induce cell cycle arrest, increase caspases production and induce apoptosis in human cancerous cells through double dual inhibition of topoisomerase I/II^14–16^. Moreover, the 9-bromo/methoxy derivatives of 1*H*-benzo[*f*]chromene (**B**) cause a cell cycle stops at the G_2_/M, S, and S-G_2_/M phases, increase caspases production, and finally cause intrinsic and extrinsic apoptotic cell death^17,18^. Finally, 1*H*-benzo[*f*]chromene (**C**) derivatives have high hAChE properties^19^, and tetrazolyl derivatives (**D**) act as an anticancer agent^20^, as presented in (Fig. 3).

The multidrug resistance (MDR) is a common phenomenon of resistance to chemotherapeutic drugs manifested by malignant tumors during chemotherapy, which can be simply described as the inability of anti-tumor drugs to accumulate in intracellular to sufficient concentrations due to significant efflux effect mediated by ATP-dependent processes^21,22^. This transport technique is directly related to the overexpression of ATP binding cassette (ABC) transporters, a kind of membrane proteins, such as *P*-glycoprotein (*P*-gp, ABCB1)^23^, Breast cancer resistance protein (BCRP, ABCG2), and Multidrug Resistance associated protein 1 (MRP1, ABCC1)^24,25^. Among ABC transporter family, *P*-glycoprotein was identified as the first member related to MDR. A mandatory option for clinical treatment of malignant tumors is chemotherapy; however various frontline drugs are susceptible to *P*-gp-mediated efflux, such as doxorubicin, paclitaxel, daunorubicin and vincristine, among many others^26^. Also, it is well known that *P*-gp is an ideal target for reversing MDR.

This study's specific aims and objectives are to further our ongoing efforts to synthesise compounds based on pyrans that have anticancer activity^27–49^. In this work, we examine the anti-proliferative activity of the *β*-enaminonitrile at C-1/9 positions (phenyl and hydroxyl groups).

**Figure 4:**
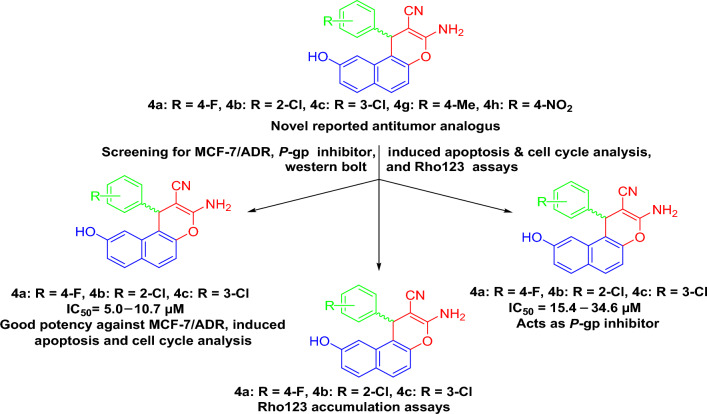
Study rationale analysis of antitumor activities, MCF-7/ADR, *P*-gp inhibitor, apoptosis and cell cycle analysis and Rh123 results of target compounds.

To reach aim, cancerogenic cell lines (PC-3, SKOV-3, and HeLa) were used to study the anti-proliferative potency. The superior cytotoxic compounds **4a–c, 4g,** and **4h** were then submitted to cancerogenic (MCF-7/ADR), non-cancerogenic cell lines (HFL-1, WI-38) and tested as *P*-gp inhibitors. In addition, Rho123 accumulation tests demonstrated that compounds **4a-c** effectively inhibited *P*-pg and efflux function, while **4a-c** compounds induce apoptosis and accumulation of MCF-7/ADR cells processed in the S and G2/M phase of the cell cycle, as illustrated in (Fig. 4).

**Figure 5:**
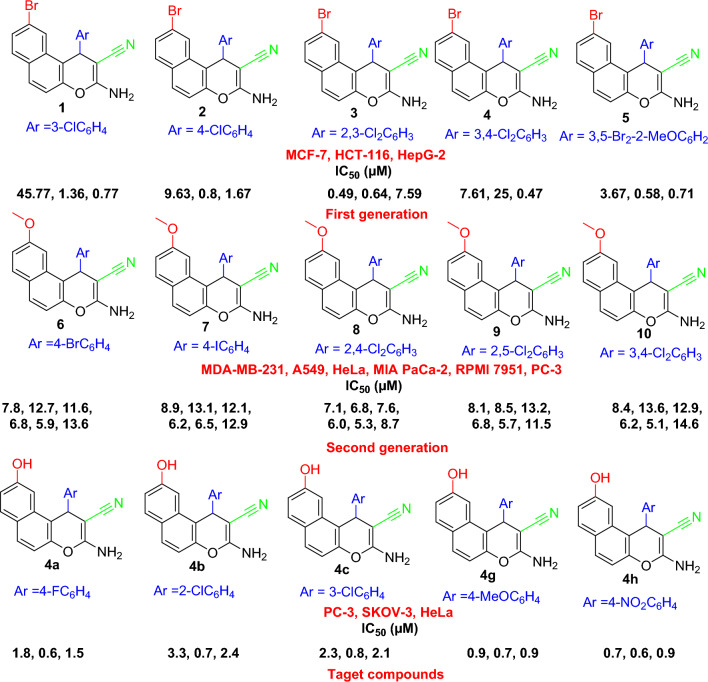
Rationale for designing target compounds.

The following comprised the logical analysis of the target compounds:

- The 9-position substitution in the scaffolding of 1*H*-benzo[*f*]chromene.
- The various aryl group substituents connected to the 1*H*-benzo[*f*]chromene at the 1-position.
- As illustrated in (Fig. 5), a comparison of the methods of the newly introduced substituent (9-OH) with the previously prepared derivatives connected to various substituent at the 9-Br (first generation)^17^, and second generation (9-OMe)^18^. Only the most recent products, **4a–c, 4g,** and **4h** were found to have demonstrated strong anti-cancer activities and to be either more or equally potent than the previously manufactured derivatives 1–10^17,18^.

## Results and discussion

### Chemistry

**Figure 6:**
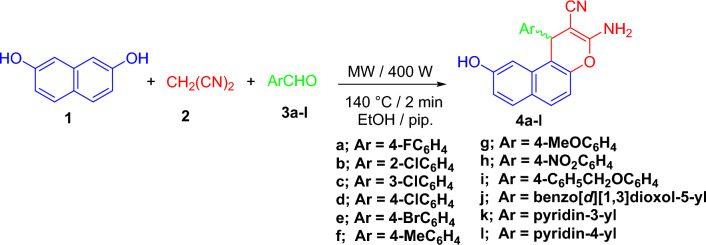
Microwave irradiation Synthesis of 3-amino-1-aryl-9-hydroxy-1*H*-benzo[f]chromene-2-carbonitrile **(4a–l)**.

A combination of naphthalene-2,7-diol (**1**), malononitrile (**2**), and suitable aryl/hetaryl aldehydes (**3**) was reacted in a piperidine ethanol solution to create *β*-enaminonitrile (**4a–l**). The reaction was conducted under Microwave irradiation conditions, with a maximum power of 400 W and a reaction time of 2 min. at 140 °C. The result was 3-amino-1-aryl-9-hydroxy-1*H*-benzo[*f*]chromene-2-carbonitriles (**4a–l**) (Fig. 6). Compound **4** forms a racemic (±) mixture and is optically inactive^50–52^.

### Spectroscopic data

The spectral data correlated with the structures. Infrared spectra of **4c,h–l** revealed absorption bands *υ* 3479–3401, 3445–3309, 3322–3207, 3226–3198 cm^−1^ for amino and hydroxyl groups, in addition to the absorption bands of the nitrile groups at *υ* 2191–2175 cm^−1^. Additionally, the ^1^H NMR spectra of **4h–l** revealed the signals of the hydroxyl, amino and methine protons in the range of *δ* 9.93–9.87, 7.11–6.90, 5.28–4.96 ppm. The ^13^C NMR spectra of **4h–l** showed resonating signals within the *δ* 38.49–36.37.94 ppm range attributed to the methine carbons. Furthermore, the MS spectra of **4h–l** and ^13^C NMR-APT spectra of **4c** and **4f** confirmed their structures (see supplementary materials S1–S21).

### Biological activity

#### In vitro cytotoxic activity

**Figure 7:**
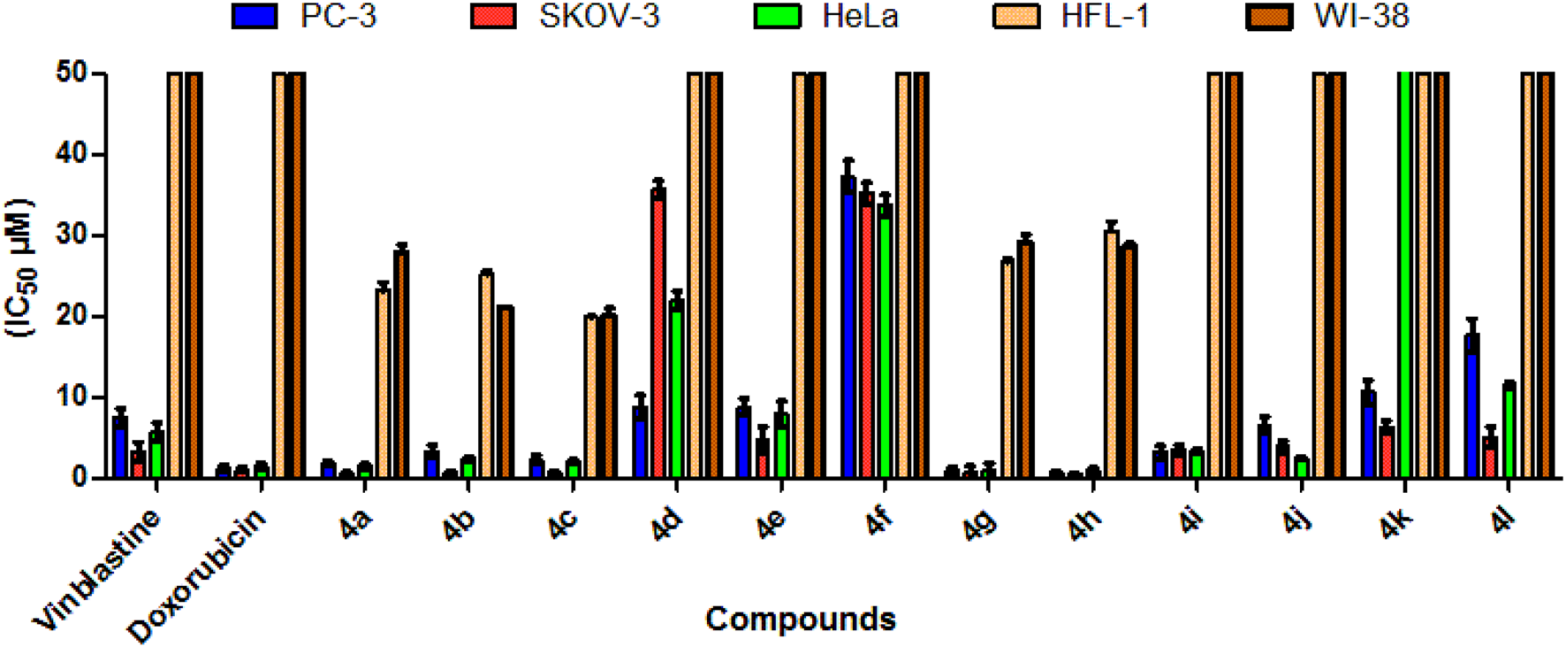
IC_50_ values are expressed in (μM) of the target compounds **4a–4l** against PC-3, SKOV-3, HeLa tumor cells and human fetal lung (HFL-1) and human diploid fibroblasts (WI-38) normal cell lines.

**Figure 8:**
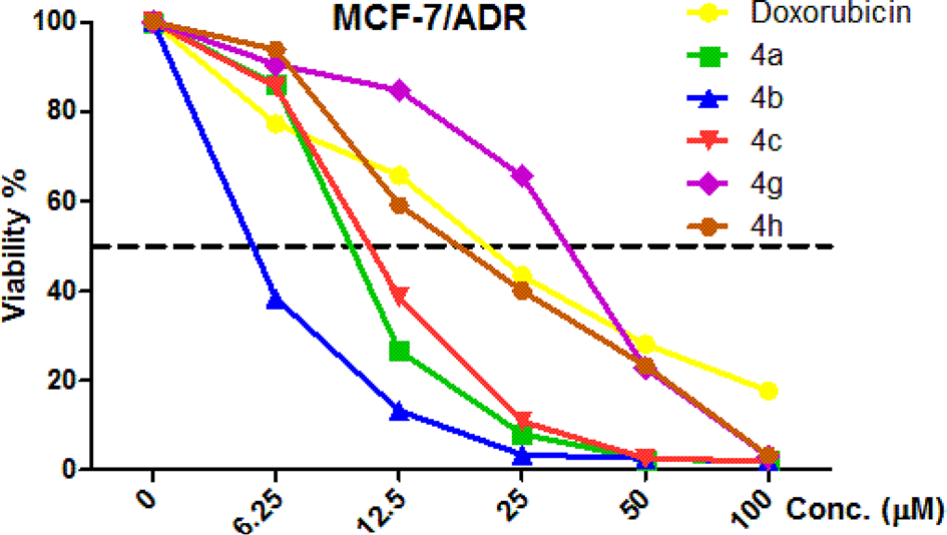
Dose dependent cytotoxicity in MCF-7/ADR cell and the effect of varying concentrations of tested compounds **4a–4c, 4g,** and **4h** on cell growth of MCF-7/ADR following exposure 24 h.

Using concentrations ranging from 0 to 100 μM against PC-3, SKOV-3, and HeLa cancerogenic cell lines, target compounds **4a–l** were assessed for their anticancer activities^53^ and compared with the commercially available Vinblastine and Doxorubicin. The results were expressed (IC_50_ μM) after 24 h of incubation (Figs. 7 and Table 1). The compounds with the greatest cytotoxic activity against the cancerous cell lines PC-3, SKOV-3, and HeLa were **4a–c**, **4g,** and **4h**. These actions were on par with or more effective than those of Doxorubicin and Vinblastine. We selected the cytotoxic active compounds **4a–c**, **4g**, and **4h** to be evaluated against MCF-7/ADR cancerogenic and HFL-1 and WI-38 non-cancerogenic cell lines (Figs. 7, 8 and Table 1).

**Table 1:** IC_50_ values of the target compounds **4a–l** against a variety cell lines.


IC_50_ µM^a^							
Cancerotoxicity					Normotoxicity		
Cpd	Ar	PC-3	SKOV-3	HeLa	MCF-7/ADR	HFL-1	WI-38
**4a**	4-FC_6_H_5_	1.8 ± 0.3	0.6 ± 0.2	1.5 ± 0.3	9.5 ± 0.3	69.8 ± 0.3	83.7 ± 1.1
**4b** **4c**	2-ClC_6_H_5_	3.3 ± 0.5	0.7 ± 0.2	2.4 ± 0.2	5.0 ± 0.1	72.0 ± 0.2	61.1 ± 0.1
	3-ClC_6_H_5_	2.3 ± 2.2	0.8 ± 0.1	2.1 ± 0.2	10.7 ± 0.3	56.5 ± 1.2	58.2 ± 1.1
**4d**	4-ClC_6_H_5_	8.8 ± 0.7	35.6 ± 0.4	21.9 ± 1.1	–	–	–
**4e**	4-BrC_6_H_5_	8.8 ± 1.2	4.8 ± 1.1	7.9 ± 1.6	–	–	–
**4f**	4-MeC_6_H_5_	37.2 ± 1.3	35.2 ± 1.3	33.7 ± 1.3	–	–	–
**4g**	4-MeOC_6_H_5_	0.9 ± 0.4	0.7 ± 0.8	0.9 ± 1.5	30.5 ± 0.7	77.5 ± 0.1	84.5 ± 1.1
**4h**	4-NO_2_C_6_H_5_	0.7 ± 0.4	0.6 ± 0.1	0.9 ± 0.4	19.2 ± 0.7	84.9 ± 0.1	79.4 ± 0.1
**4i**	4-PhCH_2_OC_6_H_5_	3.2 ± 1.0	3.5 ± 0.3	3.3 ± 0.3	–	–	–
**4j**	benzo[d][1,3]dioxol-5-yl	6.6 ± 0.5	3.9 ± 0.6	2.5 ± 0.2	–	–	–
**4k**	pyridin-3-yl	10.6 ± 0.5	6.3 ± 0.8	76.4 ± 0.7	–	–	–
**4l**	pyridin-4-yl	17.7 ± 0.7	5.1 ± 0.8	11.5 ± 0.9	–	–	–
**A**	–	7.5 ± 1.3	3.2 ± 1.2	5.7 ± 1.1	–	–	–
**B**	–	1.3 ± 0.3	1.1 ± 0.2	1.5 ± 0.3	18.6 ± 0.2	–	–

^a^IC_50_ values expressed in (μM) as the mean values of triplicate wells from at least three experiments and are reported as the mean ± standard error. **A** = Vinblastine, **B** = Doxorubicin.

Table 1 indicated that **4h** and **4g** (IC_50_ = 0.7 ± 0.4 and 0.9 ± 0.4 μM) were the most potent derivatives, being 10.7, 8.3 stronger and more effective than Vinblastine (IC_50_ = 7.5 ± 1.3 μM) and 1.6, 1.4 times more potent than Doxorubicin (IC_50_ = 1.3 ± 0.3 μM), while **4a, 4c, 4i,** and **4b** (IC_50_ = 1.8 ± 0.3, 2.3 ± 2.2, 3.2 ± 1.0 and 3.3 ± 0.5 μM) have superior potent action against the PC-3 cell line than Vinblastine (IC_50_ = 7.5 ± 1.3 μM). With regard to activity against SKOV-3 cells, **4a, 4h, 4b, 4g,** and **4c** were the stronger analogs in this study, with IC_50_ values of 0.6 ± 0.2, 0.6 ± 0.1, 0.7 ± 0.2, 0.7 ± 0.8 and 0.8 ± 0.1 μM (Fig. 8). They showed 5.3, 5.3, 4.6, 4.6, 4 times more potency than Vinblastine (IC_50_ = 3.2 ± 1.2 μM) and 1.8, 1.8, 1.6, 1.6, 1.4 times more potency than Doxorubicin (IC_50_ = 1.1 ± 0.2 μM). Besides, cytotoxicity evaluation in HeLa cell line revealed that compounds **4g, 4h, 4a, 4c,** and **4b** displayed good activities against HeLa cell lines with IC_50_ 0.9 ± 1.5, 0.9 ± 0.4, 1.5 ± 0.3, 2.1 ± 0.2 and 2.4 ± 0.2, respectively, with regard to Vinblastine (IC_50_ = 5.7 ± 1.1 μM) and Doxorubicin (IC_50_ = 1.5 ± 0.3 μM). Also, compounds **4a–4c, 4g,** and **4h** were weakly inactive against non-cancerogenic cells (HFL-1, WI-38), with an IC_50_ ranging from 56.5 to 84.9 μM. As well, compounds **4b, 4a,** and **4c** have perfect potency against MCF-7/ADR cells with IC_50_ = 5.0 ± 0.1, 9.5 ± 0.3, 10.7 ± 0.3 μM, respectively, with regard to Doxorubicin (IC_50_ = 18.6 ± 0.3 μM), while compounds **4g** and **4h** are inactive against MCF-7/ADR cell. Finally, the rest of the molecules showed moderate-to-fair potency against the cancerogenic cells regards to reference drugs.

#### *P*-glycoprotein-mediated multidrug resistance

**Figure 9:**
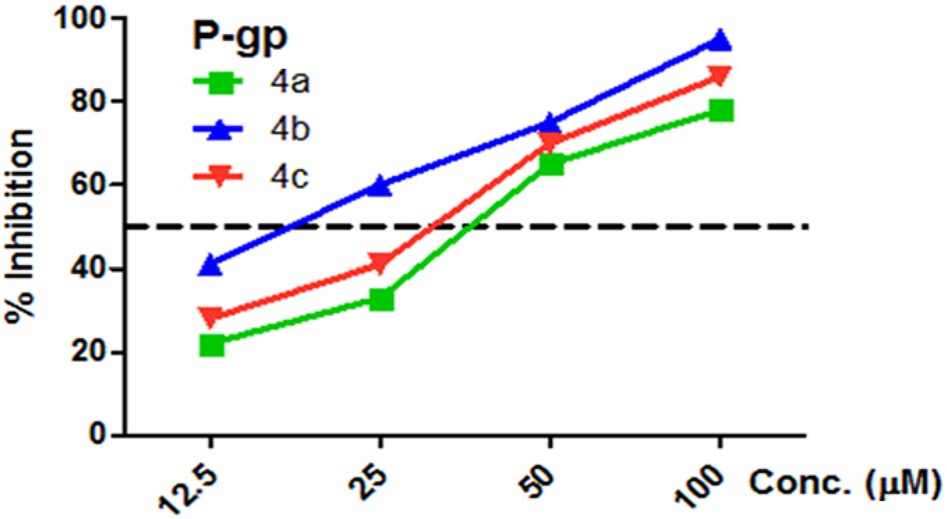
Inhibition of *P*-gp content in the cell lysate of MCF-7/ADR using varying conc. (12.5–100 µM) of tested compounds **4a–c** follows exposure 48 h. as determined by ELISA.

The* P*-gp macromolecule imparts multidrug resistance (MDR) on the cancers cell^54^ and malignant tumor clinical therapeutics^26^. The active compounds **4a–c** against MCF-7/ADR cell lines with 4-F, 2-Cl, and 3-Cl substituents were tested as *P*-gp inhibitors and have shown a good strength against *P*-gp-mediated MDR in MCF-7/ADR with IC_50_ ranging 15.4–34.7 μM compared with Doxorubicin (IC_50_ = 50.9 μM), as shown in Table 2 and Fig. 9.

**Table 2:** *P*-gp inhibitory potential (IC_50_ values) based on *P*-gp content in MCF-7/ADR cell lysate and rhodamine 123 accumulation assay.

Cpd.	MCF-7/ADR IC_50_ (µM)	*P*-gp IC_50_ (µM)	Rho123 IC_50_ (µM)
4a	9.5 ± 0.3	34.7	30.9
4b	5.0 ± 0.1	15.4	13.3
4c	10.7 ± 0.3	27.3	21.5
Doxorubicin	18.6 ± 0.2	50.9	–
Verapamil	–	–	14.3

These results show that only compounds **4a–c** have a superior effect on the *P*-gp which has high potency by reversing the MDR in MCF-7/ADR. In addition, the influence of our synthesized active compounds **4a–c** has been tested for its possible inhibitor of *P*-gp activity using a Rhodamine 123 Accumulation Assay (Rhodamine Competitive ELISA Kit). As illustrated in Table 2. The data presented in Table 2 (IC_50_) of Rhodamine 123 for the assessment of functional inhibition *P*-gp of compounds **4a–c**, ranging from 13.3 to 30.9 µM compared to the reference drug Verapamil (14.3 µM). The compounds **4a–c** reduced* P*-gp expression as well as its function, which had an impact on the recovery of sensitivity to MCF-7/ADR cells.

#### Cell cycle arrest

*P*-gp breast cancer resistance proteins (BCRP) stop the impact of anticancer drugs on the cell cycle arrest^55^. Cancer cells undergo unscheduled cell divisions by the down regulation of the four cell cycle stages (G1, S, G2, and M). As a result, the development of anti-cancer agents targeting cell cycle arrest represents an important therapeutic intervention^56,57^.

**Figure 10:**
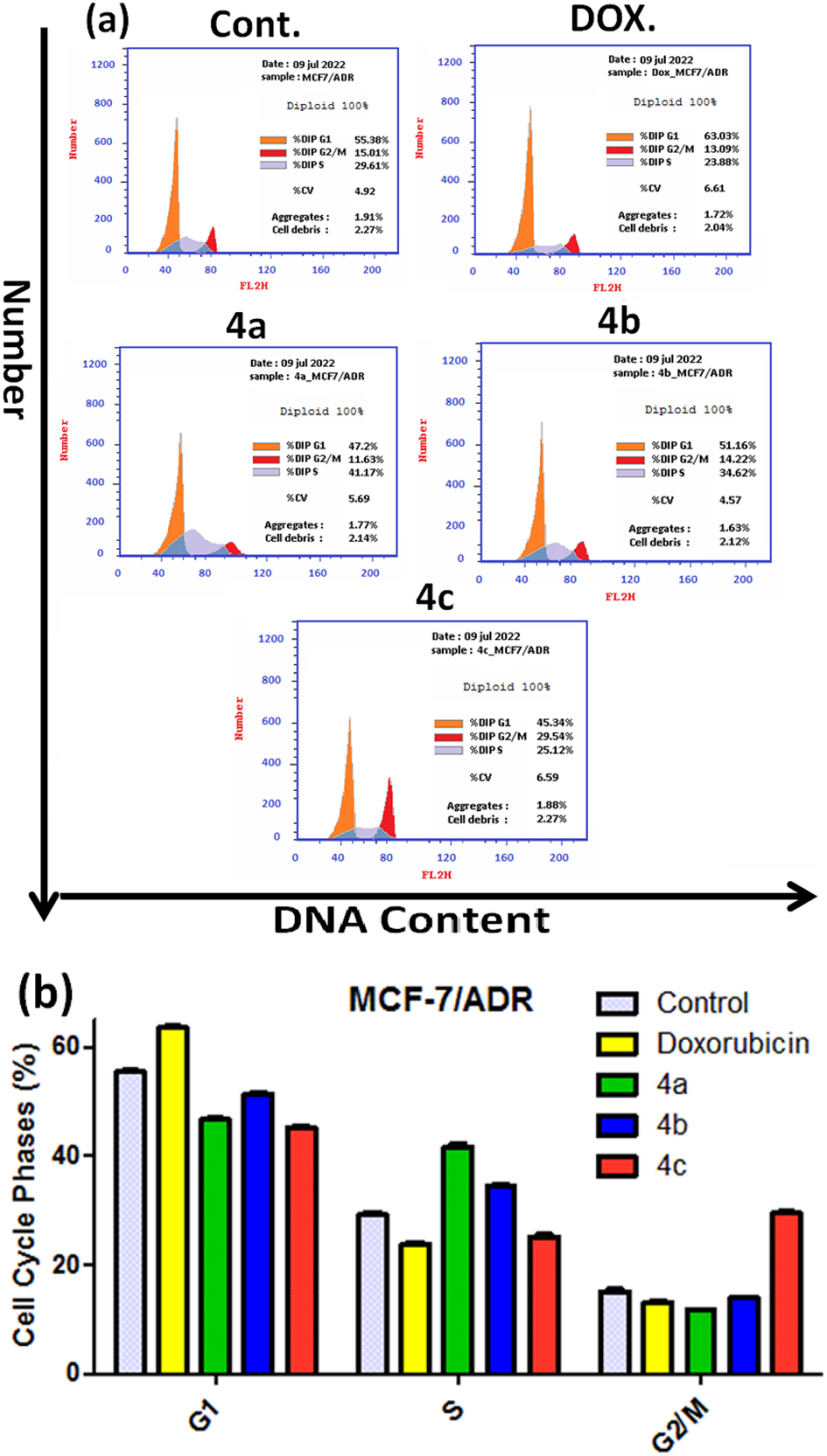
Effects of compounds **4a–c,** on the cell cycle phases of MCF-7/ADR cells. (**a**) Histograms of the DNA content. (**b**) The percentage of MCF-7/ADR cells in the G1, S, and G2/M phases. The data are expressed as the mean ± SD of three independent experiments in triplicate.

The most powerful recently synthesized substances **4a–c** on regulating cell cycle progression of MCF-7/ADR cells was analyzed by the flow cytometry, exploiting the FACS Calibers (Becton Dickinson). The typical cells distribution histogram of the stained DNA showed the spread of cells along the various cycle stages (Fig. 10a).

The MCF-7/ADR cancer cells were treated with each derivative at its IC_50_ values for 24 h. Cell cycle progression results of compounds **4a** and **4b** show cell cycle arrest at S phase with 41.17% and 34.62% respectively compared to 29.61% of the negative untreated control. On the other hand, compound **4c** causes cell cycle arrest at G2/M in 29.54% of the treated cell compared to 15.01% of the untreated cells. Comparing the treated control cells to our findings, there was a significant drop in the proportion of the cell in the G1 phase contrary to the Doxorubicin results (Fig. 10b). The cell cycle evaluation presented that the tested derivatives significantly arrested the MCF-7/ADR cancer cells at S and G2/M phases.

#### Cell apoptosis

**Figure 11:**
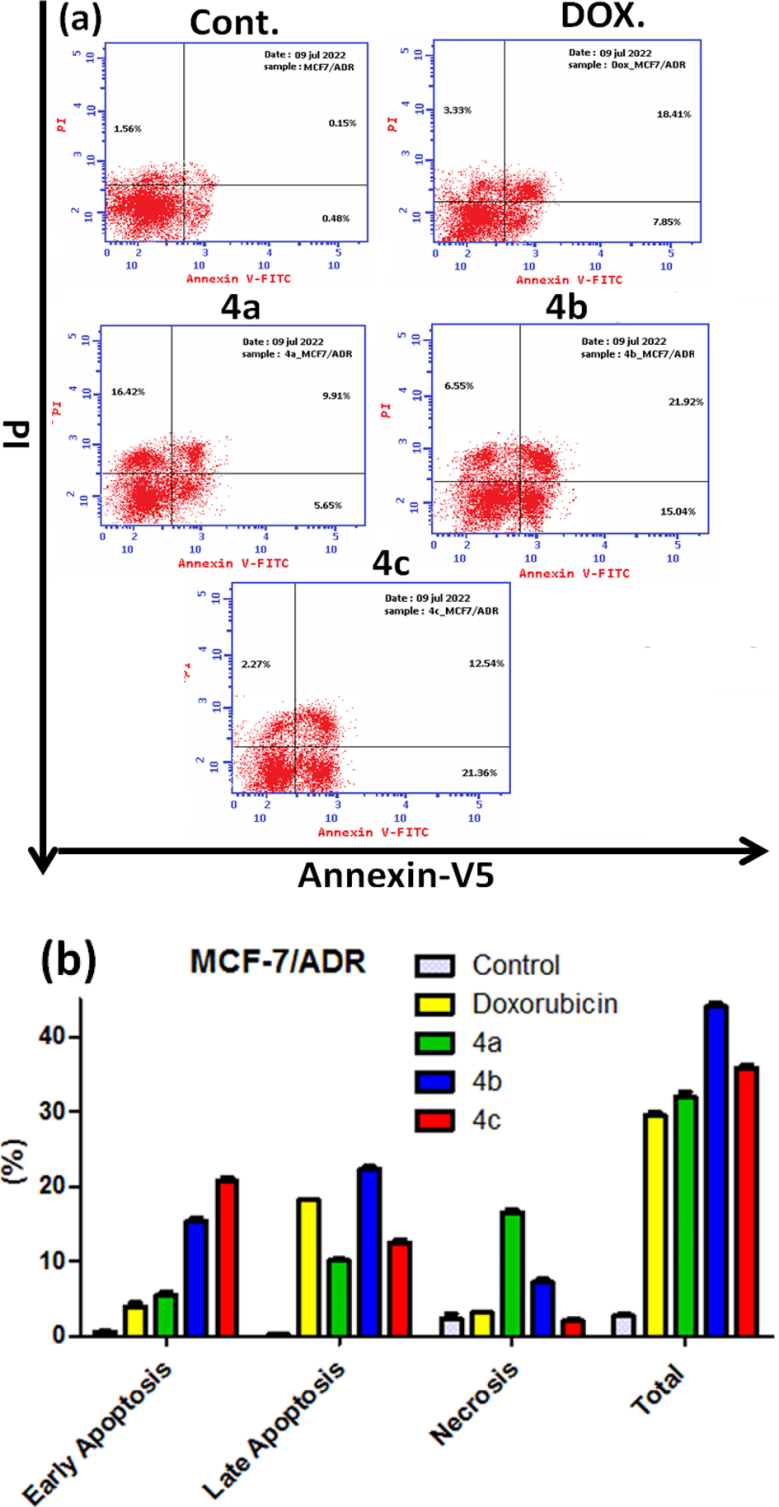
Apoptosis of MCF-7/ADR cells treated with compounds **4a–c**. (**a**) The dot plot of the Annexin V/PI-stained cells, treated with the indicated drugs. (**b**) The apoptosis percentage of MCF-7/ADR cells. The data are expressed as the mean ± SD of three independent experiments in triplicate.

*P*-gp inhibits apoptosis by preventing the release of cytochrome c which is mediated by the intrinsic mitochondrial pathway^58^. Also, blocking cell cycle progression, inducing apoptosis or the combined effect of both are one of the indicated mechanisms for the cytotoxic effect of the chemotherapeutic drugs^59^. Therefore, further assessment of the pivotal relationship between the newly synthesized MCF-7/ADR anticancer compounds and apoptosis was measured by the means of the Annexin V/PI double staining flow cytometric assay^60^. The representative dot plots of the double-stained MCF-7/ADR cells after treatment with the diverse examined compounds were displayed in (Fig. 11a).

All the tested compounds **4a-c** showed early as well as late apoptosis, with highest total apoptosis percentage (31.98%), (43.51%), and (36.17%) respectively compared to 29.59% of Doxorubicin reference drug. Moreover, increased necrosis effects were noticed only with compounds **4a** (16.42%) as compared with Doxorubicin (3.33%) (Fig. 11b). Our results proposed that the induction of MCF-7/ADR cytotoxicity occurs through mechanisms associated with apoptosis with no obvious negative effects of the *P*-gp.

#### Structure–activity relationship (SAR) study

The SAR study deals with aryl and hydroxyl groups at 1-positon and 9-positon of benzochromene moieties and their effects on the antitumor activities of the target molecules. The order of potency of **4a–l** was dependent on the type of the substitution on the phenyl group, and the potency was decreased in the order of 4-NO_2_ > 4-MeO > 4-F > 3-Cl > phCH_2_O > 2-Cl > benzo[*d*][1,3]dioxol-5-yl > 4-Cl > 4-Br > pyridin-3-yl > pyridin-4-yl > 4-Me for the PC-3 cancer cells with IC_50_ = 0.9–37.2 μM, indicating that grafting a lipophilic electron-withdrawing substituent such as nitro or halogen is more valuable for the activity than an electron-donating substituent such as methyl, methoxy, benzyloxy or hetaryl group. Further study the potency of **4a–l** against SKOV-3 cancer cells revealed that the anti-proliferative activities were decreased in the order of 4-NO_2_ > 4-F > 2-Cl > 4-MeO > 3-Cl > phCH_2_O > benzo[*d*][1,3]dioxol-5-yl > 4-Br > pyridin-4-yl > pyridin-3-yl > 4-Me > 4-Cl with IC_50_ = 0.6- 35.6 μM, it is suggested that incorporation of a lipophilic electron-withdrawing substituent is preferable to electron-donating groups for an activity. On the other hand, compounds **4g, 4h, 4a, 4c,** and **4b** offered good potency against the HeLa cell lines with an IC_50_ = 0.9–2.4 μM, hinting that nitro, methoxy and halogens substituents incorporation may be advantageous. Furthermore, compounds **4a–c, 4g,** and **4h** had weak activity against non-cancerogenic cells (HFL-1 and WI-38) with an IC_50_ = 56.5–84.9 μM. Besides, compounds **4b, 4a,** and **4c** have good potency against MCF-7/ADR cells with IC_50_ = 5.0 ± 0.1, 9.5 ± 0.3, 10.7 ± 0.3 μM, respectively, with regard to Doxorubicin (IC_50_ = 18.6 ± 0.3 μM), indicated that, for the activity, grafting a lipophilic, moderate-sized electron-withdrawing substituent such as 2-chloro, 3-chloro, or 4-fluoro on the 1-position is more advantageous than grafting an electron-donating or electron-withdrawing substituent such as 4-OMe or 4-NO_2_. Finally, the remaining compounds exhibited moderate-to-fair cytotoxic activities against the cancerogenic cells with regard to the reference drugs.

#### Molecular docking

The molecular-docking experiment was applied to examine the potential interactions of compounds **4a–c** against *P*-gp. Theoretical modeling was utilized, with the (PDB: code 3G60) serving as the basis for the experiments^61^. Human *P*-gp was generated using (I-TASSER) and optimized using the (AMBER) force field^62^. The model was identical to the experimental mouse structure described in the protein data bank, according to a generated Ramachandran diagram. Then (I-TASSER) was used to predict *P*-gp (PDB: code 3G60) mouse crystal structure, which was used as a rigid object in the docking study. The obtained data from the docking procedure provided insight into the appropriate complex geometry for ATP hydrolysis. This approach provided a deeper understanding of the mechanisms of compounds action against *P*-gp and may help in the creation of more potent treatments for disorders brought on by drug resistance. We conducted a redocking experiment using (QZ59-RRR) as a cyclic peptide reference which bound to *P*-gp (PDB; code 3G60) and compared it to (QZ59-RRR) original geometry to confirm the accurateness of the docking experiment. QZ59-RRR which is the reference inhibitor in the crystal *P*-gp macromolecule was docked with high precision into the experimentally obtained mouse *P*-gp structure and resulted in an RMSD value of 1.78 Å, these results imply that the docking analysis was precise and trustworthy. The binding free energies ΔG obtained from the redocking experiment utilizing Glide's module®^63,64^ are shown in (Table 3).

**Table 3:** The binding affinity of **4a-l** and QZ59-RRR ligands in (kcal/mol) against *P*-gp.

Cpd.	ΔG	rmsd	H.B	Int	E_ele		ΔG	rmsd	H.B	Int	E_ele
**4a**	− 10.61	1.30	− 33.93	− 22.38	− 9.12	**4h**	− 9.94	1.76	− 40.86	− 18.04	− 7.77
**4b**	− 10.79	0.98	− 35.14	− 18.71	− 7.26	**4i**	− 9.84	3.55	− 33.12	− 15.69	− 7.74
**4c**	− 11.04	1.28	− 7.13	− 21.49	− 9.04	**4j**	− 10.08	1.74	− 26.39	− 19.90	− 9.08
**4d**	− 10.49	1.34	− 36.75	− 22.37	− 9.30	**4k**	− 9.05	1.16	− 48.66	− 8.52	− 7.69
**4e**	− 10.48	1.36	− 35.91	− 22.37	− 9.57	**4l**	− 7.93	1.52	− 48.06	− 21.05	− 7.64
**4f**	− 9.00	1.95	− 38.40	− 22.24	− 9.42	**QZ59**	− 8.57	2.62	101.30	− 24.46	− 8.77
**4g**	− 10.43	1.35	− 38.66	− 24.46	− 8.52						

The crystal structures of the original inhibitors were adapted to fit properly into their respective binding sites. QZ59-RRR established identical hydrogen bonding with Gln721 and Ser725 and capped the binding pocket in PDB code 3G60. Like the original inhibitor, all active compounds **4a–l** successfully docked onto the active sites (Table 3).

Table 3 indicates that, compounds **4a–c** which was the most potential anti-proliferative compounds, showed greater efficiency of binding against (QZ59-RRR). Among investigated compounds and reference inhibitors, compound **4c** displayed the highest ∆G = − 11.04 kcal mol.^−1^, other two active compounds, **4a** and **4b**, likewise showed higher G values than QZ59-RRR, and as shown in Table 3, which organized in reducing order as **4c** < **4b** < **4a** < (QZ59-RRR).

**Figure 12:**
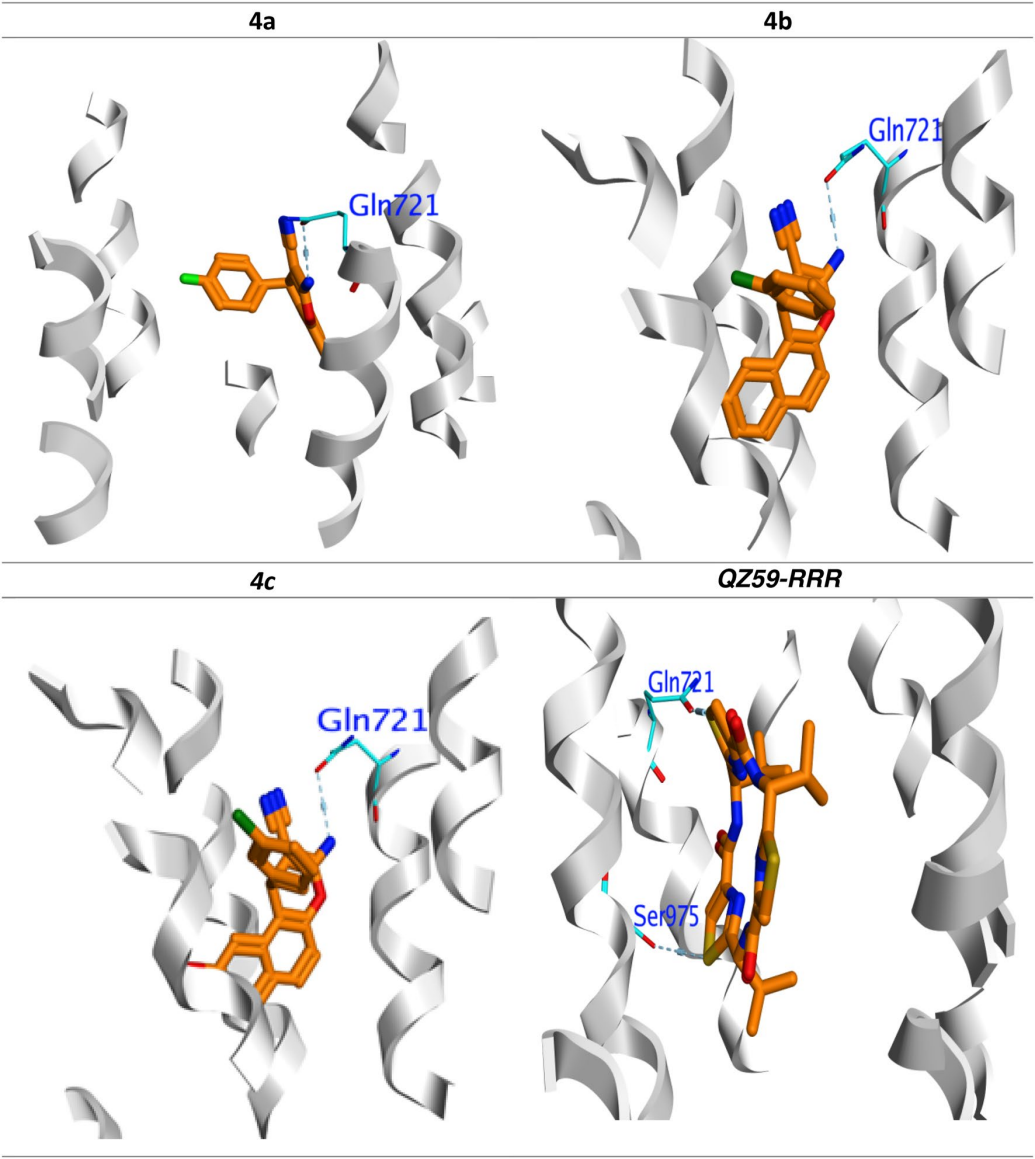
Binding mode of most active compounds and QZ59-RRR into *P*-gp (PDB: 3G60). H-bonding represented in blue lines.

These compounds **4a, 4b** and **4c** occupied the binding pocket in the same manner as (QZ59-RRR) by engaging Gln721 (Fig. 12). These findings can explain their promising activity toward inhibition of the *P*-gp with (IC_50_ = 34.7 μM, 15.4 μM and 27.3 μM). Compound **4b** showed the highest hydrogen bond interaction H.B. = − 35.14 kcal mol.^−1^ compared to other compounds **4a** and **4c** which explain the highest biological activity against *P*-gp.

#### Physicochemical parameters profiles

**Figure 13:**
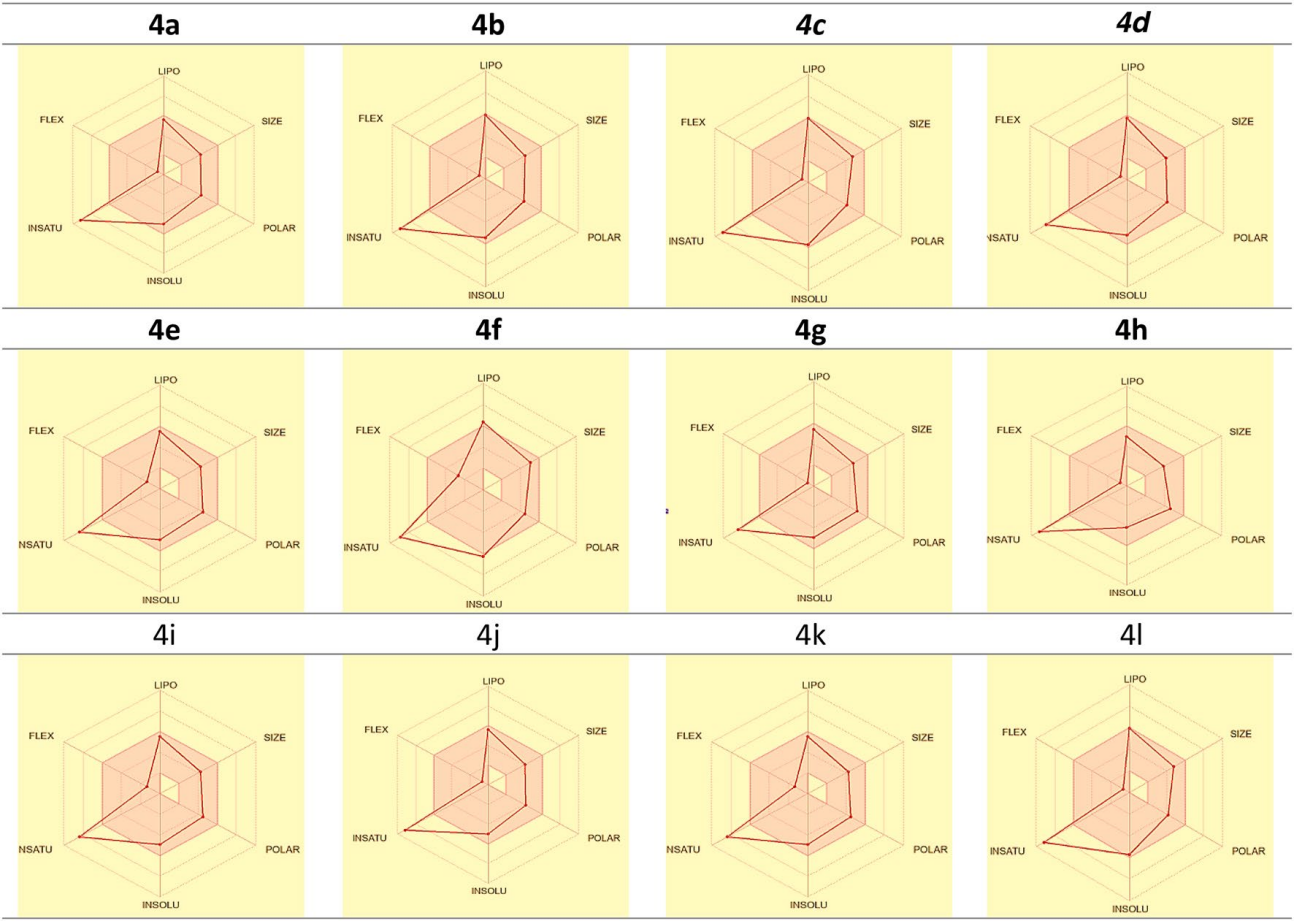
Charts for Bioactivity radar for **4a–l;** where FLEX, flexibility; LIPO, lipophilicity; INSATU, instauration, and INSOLU, insolubility.

**Figure 14:**
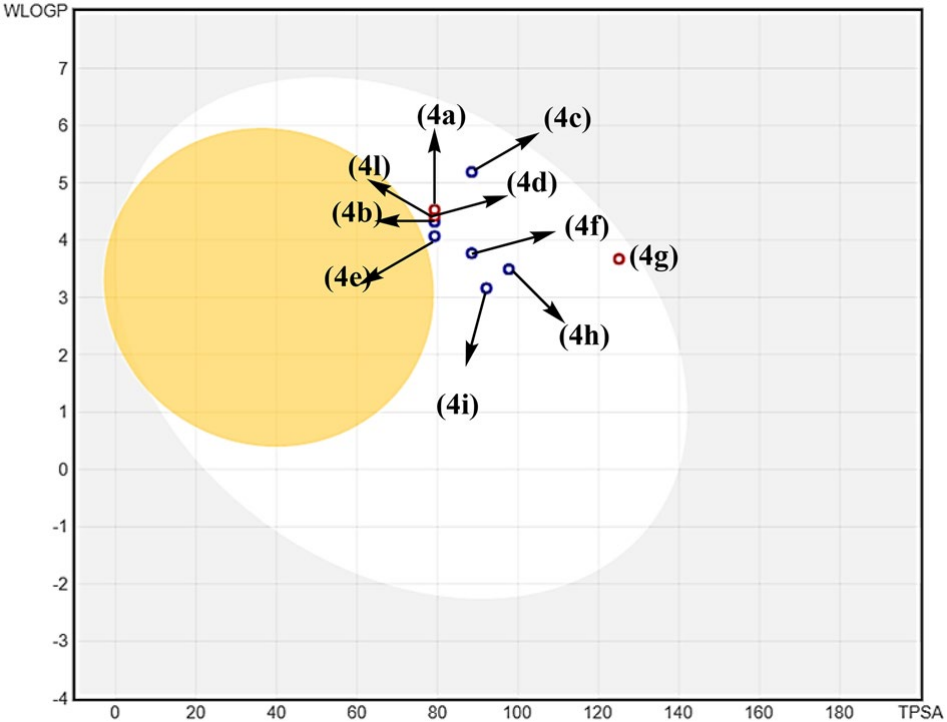
Boiled-egg chart for **4a–l**.

The physicochemical parameters of most active hybrids **4a-l** were examined using Swiss-ADME^65^ including (lipophilicity, solubility, pharmacokinetics, drug likeness, and medicinal chemistry) and represented in (Figs. 13, 14 and Tables 4). These parameters are crucial in determining the potential of these compounds as drug candidates.

**Table 4:** Prediction of ADMET for tested **4a-e** compounds.

Molecule	**4a**	**4b**	**4c**	**4d**	**4e**
MW	332.33	348.78	348.78	393.23	328.36
#Heavy atoms	25	25	25	25	25
#Aromatic heavy atoms	16	16	16	16	16
Fraction Csp3	0.05	0.05	0.05	0.05	0.1
#Rotatable bonds	1	1	1	1	1
#H-bond acceptors	4	3	3	3	3
#H-bond donors	2	2	2	2	2
MR	91.36	96.41	96.41	99.1	96.36
TPSA	79.27	79.27	79.27	79.27	79.27
iLOGP	2.36	2.39	2.55	2.65	2.58
XLOGP3	4.29	4.82	4.82	4.88	4.55
WLOGP	4.32	4.42	4.42	4.53	4.07
MLOGP	2.71	2.83	2.83	2.94	2.56
Silicos-IT Log P	3.61	3.83	3.83	3.87	3.71
Consensus Log P	3.46	3.66	3.69	3.77	3.49
ESOL Log S	− 5.01	− 5.45	− 5.45	− 5.76	− 5.15
ESOL Solubility (mg/ml)	3.24E−03	1.25E−03	1.25E−03	6.83E−04	2.32E−03
ESOL Solubility (mol/l)	9.76E−06	3.58E−06	3.58E−06	1.74E−06	7.08E−06
ESOL Class	Moderately soluble	Moderately soluble	Moderately soluble	Moderately soluble	Moderately soluble
Ali Log S	− 5.67	− 6.22	− 6.22	− 6.28	− 5.94
Ali Solubility (mg/ml)	7.14E−04	2.11E−04	2.11E−04	2.06E−04	3.79E−04
Ali Solubility (mol/l)	2.15E−06	6.06E−07	6.06E−07	5.25E−07	1.16E−06
Ali Class	Moderately soluble	Poorly soluble	Poorly soluble	Poorly soluble	Moderately soluble
Silicos-IT LogSw	− 6.3	− 6.63	− 6.63	− 6.83	− 6.41
Silicos-IT Solubility (mg/ml)	1.65E−04	8.18E−05	8.18E−05	5.79E−05	1.27E−04
Silicos-IT Solubility (mol/l)	4.97E−07	2.34E−07	2.34E−07	1.47E−07	3.85E−07
Silicos-IT class	Poorly soluble	Poorly soluble	Poorly soluble	Poorly soluble	Poorly soluble
GI absorption	High	High	High	High	High
BBB permeant	No	No	No	No	No
*P*-gp substrate	Yes	No	No	No	Yes
CYP1A2 inhibitor	Yes	Yes	Yes	Yes	Yes
CYP2C19 inhibitor	Yes	Yes	Yes	Yes	Yes
CYP2C9 inhibitor	Yes	Yes	Yes	Yes	Yes
CYP2D6 inhibitor	No	No	No	No	No
CYP3A4 inhibitor	No	Yes	No	No	No
log Kp (cm/s)	− 5.28	− 5.01	− 5.01	− 5.23	− 5.07
Lipinski #violations	0	0	0	0	0
Ghose #violations	0	0	0	0	0
Veber #violations	0	0	0	0	0
Egan #violations	0	0	0	0	0
Muegge #violations	0	0	0	0	0
Bioavailability Score	0.55	0.55	0.55	0.55	0.55
PAINS #alerts	0	0	0	0	0
Brenk #alerts	0	0	0	0	0
Leadlikeness #violations	1	1	1	2	1
Synthetic accessibility	3.71	3.75	3.73	3.78	3.83

The active hybrids had a topological polar surface area (TPSA) ranging from 79.27 to 125.09 Å^2^. The lipophilicity descriptor (log Po/w) has critical importance for pharmacokinetics drug discovery^66^. The Swiss-ADME displayed the predictive XLOGP3^67^ and WLOGP^68^ models. The tested compounds **4a–l** displayed values in rang 4.29–5.65 for XLOGP3 and 3.67–5.19 for WLOGP, respectively. Furthermore, all tested hybrids **4a–l** represented water solubility log S value less than 5 (ESOL model)^69^, which is considered favorable for bioavailable drugs. Besides, Lipinski's rule of five, which is a set of guidelines used to assess the likelihood that a compound will be orally bioavailable. According to Lipinski’s rule^70^, orally active drugs should have a molecular weight ≤ 500, no more than 5 H-bonding donors, no more than 10 H-bonding acceptors, and a log Po/w value ≤ 5. The data from (Table 4) suggest that the tested **4a–l** compounds meet these criteria. The graphical estimations for compounds for gastrointestinal absorption (HIA) and blood–brain barrier (BBB) permeability using the Boil-Egg method were discussed (Fig. 14). The Boil-Egg fingerprint calculates the polarity (TPSA) and lipophilicity (WLOGP) of small molecules to estimate their potential for absorption and permeability^71^. According to the result Table 4, all of the tested compounds **4a-I** showed high gastrointestinal absorption with no permeability to the BBB^72^. This suggests that compounds **4a-I** have potential as drug candidates that can be easily absorbed by the gastrointestinal tract while avoiding potential BBB permeability. We note that **4a-I** compounds may be substrates for *P*-glycoprotein, which could reduce their absorption and penetration in the brain. However, this may be beneficial in avoiding potential side effects such as depression or drowsiness in the central nervous system. Overall, we suggest that **4a-I** compounds may be have potential as promising drug candidates based on their physicochemical properties and estimated bioavailability.

## Experimental section

### Materials and equipment’s

All chemicals were purchased from Sigma-Aldrich Chemical Co. (Sigma-Aldrich Corp., St. Louis, MO, USA). All the melting points were measured with a Stuart Scientific Co. Ltd apparatus, which means they are uncorrected. The IR spectra were recorded on a KBr disc on a Jasco FT/IR 460 plus spectrophotometer. The ^1^H/^13^C (500/125 MHz) NMR and ^13^C NMR-APT spectrum (125 MHz) spectra were measured on a BRUKER AV 500 MHz spectrometer in DMSO-d6, using tetramethylsilane (TMS) as an internal standard. The Microwave apparatus utilized is Milestone Sr1, Microsynth. The mass spectra were determined on a Shimadzu GC/MS-QP5050A spectrometer. The elemental analysis was carried out at the Regional Centre for Mycology and Biotechnology (RCMP), Al-Azhar University, Cairo, Egypt, and the results were within ± 0.25%. The reaction courses and product mixtures were routinely monitored by thin layer chromatography (TLC) on silica gel precoated F_254_ Merck plates.

### General procedure for synthesis of 1*H*-benzo[*f*]chromene derivatives (4a-l)

In an ethanol solution (30 ml), a reaction mixture containing naphthalene-2,7-diol (**1**) (0.01 mol), malononitrile (**2**) (0.01 mol), various aromatic aldehydes (**3a–l**), and piperidine (0.5 ml) was heated for two minutes at 140 °C under Microwave irradiation conditions. Upon the completion of the reaction, the reaction mixture was allowed to cool down to room temperature. The precipitated solid was then removed by filtering, cleaned with methanol, and separated from the ethanol/benzene mixture. The physical and spectral data of compounds **4a–l** are as follows:

#### 3-Amino-1-(4-fluorophenyl)-9-hydroxy-1H-benzo[f]chromene-2-carbonitrile (4a)

Yellow needles, yield 94%, m.p. 272–272 ℃ (Literature procedure, reflux condition, yield 92%; m.p. 274–276 °C^73^).

#### 3-Amino-1-(2-chlorophenyl)-9-hydroxy-1H-benzo[f]chromene-2-carbonitrile (4b)

Buff crystals; yield 86%, m.p. 262–263 ℃ (Literature procedure, reflux condition, yield 75%; m.p. 260–262 °C^74^).

#### 3-Amino-1-(3-chlorophenyl)-9-hydroxy-1H-benzo[f]chromene-2-carbonitrile (4c)

Yellow crystals; yield 88%; m.p. 260–261 ^ο^C; IR (KBr) *υ* (cm^−1^): 3456, 3358, 3226, 3196 (NH_2_ & OH), 2175 (CN); ^1^H NMR *δ*: 10.03 (s, 1H, OH), 7.89–6.97 (m, 11H, Ar and NH_2_), 5.13 (s, 1H, H-1); ^13^C NMR *δ*: 160.28, 157.08, 148.46, 147.86, 133.71, 132.47, 131.21, 130.72 (C-7), 130.03, 127.14, 126.17, 125.75, 120.83, 117.73, 113.68, 113.45, 106.09, 57.58, 37.72. In ^13^C NMR-APT *δ*: 160.28 ↓, 157.08 ↓, 148.46 ↓, 147.86 ↓, 133.71 ↓, 132.47 ↓, 131.21 ↑, 130.72 ↑, 130.03 ↑, 127.14 ↑, 126.17 ↑, 125.75 ↓, 120.83 ↓, 117.73 ↓, 113.68 ↑, 113.45 ↑, 106.09 ↑, 57.58 ↓, 37.72 ↑; MS m/z (%): 350 (M^+^ + 2, 9.43), 348 (M^+^, 28.56) with a base peak at 238 (100); Anal. Calcd for C_20_H_13_ClN_2_O_2_ (359.33): C, 68.87; H, 3.76; N, 8.03. Found: C, 68.94; H, 3.82; N, 8.10%.

#### 3-Amino-1-(4-chlorophenyl)-9-hydroxy-1H-benzo[f]chromene-2-carbonitrile (4d)

Yellow powder, yield 89%, m.p. 285–286 ℃ (Literature procedure, reflux condition, yield 75%; m.p. 286–288 °C^75^).

#### 3-Amino-1-(4-bromophenyl)-9-hydroxy-1H-benzo[f]chromene-2-carbonitrile (4e)

Yellow powder, yield 89%, m.p. 285–286 ℃ (Literature procedure, reflux condition, yield 75%; m.p. 286–288 °C^76^).

#### 3-Amino-1-(4-methylphenyl)-9-hydroxy-1H-benzo[f]chromene-2-carbonitrile (4f)

Colurless crystals; yield 91%; m.p. 258–259 ^ο^C; IR (KBr) *υ* (cm^−1^): 3448, 3351, 3229, 3199 (NH_2_ & OH), 2177 (CN); ^1^H NMR *δ*: 7.82–6.88 (m, 11H, Ar and NH_2_), 5.00 (s, 1H, H-1), 2.20 (s, 3H, CH_3_); ^13^C NMR *δ*: 160.11, 156.71, 147.69, 143.00, 136.32, 132.53, 130.60, 129.69, 127.30, 125.73, 121.14, 117.55, 114.31, 113.69, 106.19, 58.83, 37.79, 20.96. In ^13^C NMR-APT *δ*: 160.11 ↓, 156.71 ↓, 147.69 ↓, 143.00 ↓, 136.32 ↓, 132.53 ↓, 130.60 ↑, 129.69 ↑, 127.30 ↑, 125.73 ↑, 121.14 ↓, 117.55 ↑, 114.31 ↓, 113.96 ↑, 106.19 ↑, 58.83 ↓, 37.79 ↑, 20.96 ↑; MS m/z (%): 328 (M^+^, 100); Anal. Calcd for C_21_H_16_N_2_O_2_ (328.36): C, 76.81; H, 4.91; N, 8.53. Found: C, 76.88; H, 4.97; N, 8.61%.

#### 3-Amino-1-(4-methoxyphenyl)-9-hydroxy-1H-benzo[f]chromene-2-carbonitrile (4g)

Colourless needles, yield 90%, m.p. 229–230 ℃ (Literature procedure, reflux condition, yield 80%; m.p. 228 °C^77^).

#### 3-Amino-1-(4-nitrophenyl)-9-hydroxy-1H-benzo[f]chromene-2-carbonitrile (4h)

Yellow crystals; yield 88%; m.p. 262–263 ^ο^C; IR (KBr) *υ* (cm^−1^): 3456, 3358, 3226, 3196 (NH_2_ & OH), 2175 (CN); ^1^H NMR *δ*: 9.92 (s, 1H, OH), 8.18, 8.17 (dd, 2H, *J* = 7.5, 7.2 Hz, Ar, H-3,5), 7.83 (d, 1H, *J* = 8.9 Hz, H-7), 7.77 (d, 1H, *J* = 8.8 Hz, H-6), 7.43, 7.42 (dd, 2H, *J* = 7.5, 7.2 Hz, Ar, H-2,6), 7.11 (bs, 2H, NH_2_), 7.10 (s, 1H, H-10), 6.98, 6.97 (dd, 1H, *J* = 8.8, 2.3 Hz, H-8), 6.93 (d, 1H, *J* = 1.0 Hz, H-5), 5.28 (s, 1H, H-1); ^13^C NMR *δ*: 160.29, 157.03, 153.34, 147.82, 146.67, 132.43, 130.77, 130.27, 128.69, 125.73, 124.65, 120.66, 117.70, 113.68, 112.91, 106.03, 57.03, 38.49; MS m/z (%): 360 (M^+^ + 1, 100); Anal. Calcd for C_20_H_13_N_3_O_4_ (359.33): C, 66.85; H, 3.65; N, 11.69. Found: C, 66.91; H, 3.72; N, 11.74%.

#### 3-Amino-1-(4-(benzyloxy)phenyl)-9-hydroxy-1H-benzo[f]chromene-2-carbonitrile (4i)

Pale yellow crystals; yield 87%; m.p. 269–279 ^ο^C; IR (KBr) *υ* (cm^−1^): 3457, 3445, 3322, 3226 (NH_2_ & OH), 2175 (CN); ^1^H NMR *δ*: 9.87 (s, 1H, OH), 7.77 (d, 1H, *J* = 8.9 Hz, H-7), 7.74 (d, 1H, *J* = 8.8 Hz, H-6), 7.43,7.40 (dd, 2H, *J* = 8.2, 6.8 Hz, Ph, H-2,6), 7.38, 7.37 (2H, dd, *J* = 8.4, 6.9 Hz, Ph, H-3,5), 7.32 (1H, t, *J* = 7.1 Hz, Ph, H-4), 7.07, 7.06 (2H, dd, *J* = 8.8, 6.9 Hz, Ar, H-2,6), 7.05 (1H, s, H-10), 7.00 (1H, d, *J* = 2.3 Hz, H-5), 6.97 (2H, dd, J = 8.7, 2.3 Hz, Ar, H-3,5), 6.93, 6.92 (1H, d, *J* = 3.3 Hz, H-8), 6.90 (bs, 2H, NH_2_), 5.02 (s, 2H, CH_2_), 4.96 (s, 1H, H-1);

^13^C NMR *δ*: 160.00, 157.52, 156.75, 147.64, 138.38, 137.53, 132.55, 130.60, 129.98, 128.90, 128.45, 128.31, 128.19, 125.71, 121.16, 117.51, 115.29, 114.42, 113.65, 106.21, 69.68, 58.62, 38.12; MS m/z (%): 421 (M^+^ + 1, 100); Anal. Calcd for C_27_H_20_N_2_O_3_ (420.46): C, 77.13; H, 4.79; N, 6.66. Found: C, 77.17; H, 4.84; N, 6.70%.

#### 3-Amino-1-(benzo[d][1,3]dioxol-5-yl)-9-hydroxy-1H-benzo[f]chromene-2-carbonitrile (4j)

Colorless crystals; yield 86%; m.p. 260–261 °C; IR (KBr) *υ* (cm^−1^): 3401, 3315, 3207, 3194 (NH_2_ & OH), 2187 (CN); ^1^H NMR *δ*: 9.89 (s, 1H, OH), 7.78 (d, 1H, *J* = 8.9 Hz, H-7), 7.75 (d, 1H, *J* = 8.8 Hz, H-6), 7.06 (d, 1H, *J* = 8.9 Hz, H-5), 7.02 (s, 1H, H-10), 6.98 (s,1H, Ar, H-2), 6.93 (bs, 2H, NH_2_), 6.81 (s, 1H, *J* = 8.0, Hz, H-8), 6.64 (s, 1H, Ar, H-5), 6.61 (s, 1H, Ar H-6), 5.96, 5.94 (d, 2H, *J* = 14 Hz, CH_2_), 4.96 (s, 1H, H-1); ^13^C NMR *δ*: 160.05, 156.80, 147.85, 147.65, 146.32, 140.13, 132.56, 130.61, 129.68, 125.69, 121.07, 120.46, 117.56, 114.21, 113.65, 108.84, 107.77, 106.21, 101.42, 58.48, 38.49; MS m/z (%): 359 (M^+^ + 1, 100); Anal. Calcd for C_21_H_14_N_2_O_4_ (358.35): C, 70.39; H, 3.94; N, 7.82. Found: C, 70.46; H, 3.99; N, 7.88%.

#### 3-Amino-9-hydroxy-1-(pyridin-3-yl)-1H-benzo[f]chromene-2-carbonitrile (4k)

Colorless crystals; yield 80%; m.p. 296–297 ^ο^C; IR (KBr) *υ* (cm^−1^): 3479, 3309, 3217, 3191 (NH_2_ & OH), 2185 (CN); ^1^H NMR *δ*: 9.93 (s, 1H, OH), 8.51 (d, 1H, *J* = 2.5 Hz, pyridine, H-4), 8.41,8.40 (dd, 1H, *J* = 4.7, 1.7 Hz, pyridine, H-5), 7.81 (d, 1H, *J* = 8.9 Hz, H-7), 7.77 (d, 1H, *J* = 9.4 Hz, pyridine, H-6), 7.44 (d, 1H, *J* = 8.0 Hz, H-6), 7.30 (d, 1H, *J* = 8.0 Hz, H-5), 7.09 (d, 1H, *J* = 8.9 Hz, H-8), 7.07 (bs, 2H, NH_2_), 6.98 (s, 1H, pyridine, H-2), 6.97 (d, 1H, H-10), 5.15 (s, 1H, H-1); ^13^C NMR *δ*: 160.27, 156.99, 148.62, 148.47, 147.84, 141.33, 135.03, 132.30, 130.77, 130.05, 125.72, 124.61, 120.83, 117.66, 113.67, 113.07, 105.92, 57.50, 36.37; MS m/z (%): 315 (M^+^ + 1, 67) with base peak at 237 (100); Anal. Calcd for C_19_H_13_N_3_O_2_ (315.33): C, 72.37; H, 4.16; N, 13.33. Found: C, 72.45; H, 4.21; N, 13.38%.

#### 3-Amino-9-hydroxy-1-(pyridin-4-yl)-1H-benzo[f]chromene-2-carbonitrile (4l)

Colorless crystals; yield 81%; m.p. 294–295 ^ο^C; IR (KBr) *υ* (cm^−1^): 3428, 3324, 3217, 3198 (NH_2_ & OH), 2191 (CN); ^1^H NMR *δ*: 9.92 (s, 1H, OH), 8.48, 8.47 (dd, 2H, *J* = 8.8,2.4 Hz, pyridine, H-3,5), 7.82 (d, 1H, *J* = 8.9 Hz, H-7), 7.77 (d, 1H, *J* = 8.8 Hz, H-6), 7.14 (d, 1H, *J* = 1.6 Hz, H-5), 7.10 (bs, 2H, NH_2_), 7.09 (s, 1H, H-10), 6.99, 6.98 (dd, 2H, *J* = 8.8, 2.3 Hz, pyridine, H-2,6), 6.92 (d, 1H, *J* = 2.0 Hz, H-8), 5.12 (s, 1H, H-1); ^13^C NMR *δ*: 160.40, 157.01, 154.08, 150.55, 147.92, 132.43, 130.75, 130.19, 125.69, 122.68, 120.72, 117.72, 113.64, 112.63, 105.94, 56.79, 38.13; MS m/z (%): 316 (M^+^ + 1, 100); Anal. Calcd for C_19_H_13_N_3_O_2_ (315.33): C, 72.37; H, 4.16; N, 13.33. Found: C, 72.31; H, 4.11; N, 13.27%.

### Biological screening

#### Cell culture

The tumor cell lines (PC-3, SKOV-3, and HeLa), resistant cell strains (MCF-7/ADR) and the normal cell lines, (HFL-1, WI-38) were obtained from the American Type Culture Collection (ATCC, Rockville, MD, USA).

#### Cytotoxicity evaluation using viability assay

The tumor cell lines were suspended in medium at concentration 5 × 104 cells well^−1^ in Corning® 96-well tissue culture plates and then incubated for 24 h. The tested compounds with concentrations ranging from 0 to 100 μM were then added into 96-well plates (six replicates) to achieve six different concentrations for each compound. Six vehicle controls with media or 0.5% DMSO were run for each 96 well plate as a control. After incubating for 24 h, the numbers of viable cells were determined by the MTT test^53^. Briefly, the media was removed from the 96 well plates and replaced with 100 μl of fresh culture RPMI 1640 medium without phenol red then 10 μl of the 12 mM MTT stock solution (5 mg of MTT in 1 ml of PBS) to each well including the untreated controls. The 96-well plates were then incubated at 37 °C and 5% CO_2_ for 4 h. An 85-μl aliquot of the media was removed from the wells, and 50 μl of DMSO was added to each well and mixed thoroughly with the pipette and incubated at 37 °C for 10 min. Then, the optical density was measured at 590 nm with the microplate reader (Sunrise, TECAN, Inc, USA) to determine the number of viable cells and the percentage of viability was calculated as [1−(ODt/ODc)] × 100% where ODt is the mean optical density of wells treated with the tested sample and ODc is the mean optical density of untreated cells. The relation between surviving cells and drug concentration is plotted to get the survival curve of each tumor cell line after treatment with the specified compound. The 50% inhibitory concentration (IC_50_), the concentration required to cause toxic effects in 50% of intact cells, was estimated from graphic plots of the dose response curve for each concentration.

#### In vitro analysis of *P*-gp content

The content of *P*-gp in the MCF-7/ADR cell lysates after incubation with varying conc. (12.5–100 µM) of tested compounds **4a–c** following exposure for 48 h. was determined using commercial human *P*-gp (Permeability Glycoprotein) ELISA Kit (MBS2506188, MyBioSource Inc., San Diego, CA, USA). Absorption was recorded at 450 nm with a Spectramax Gemini fluorescence microplate reader (Molecular Devices, Sunnyvale, CA, USA)^78^.

#### Rhodamine 123 accumulation assay

*P*-gp activity was determined by measuring intracellular accumulation of rhodamine 123 in MCF-7/ADR cells in the absence or presence of compounds **4a–c** according to commercial Rhodamine Competitive ELISA Kit (AKR-5142, Cell Biolabs Inc., San Diego, CA, USA) which provides a convenient method for the detection of total rhodamine in extracts from cells^79^. Absorbance at 450 nm of each well was measured using Spectramax Gemini fluorescence microplate reader (Molecular Devices, Sunnyvale, CA, USA). The total content of Rhodamine in each sample was determined by comparison with a Rhodamine standard curve.

#### Cell cycle assay

Cell cycle arrest and distribution were done using Propidium Iodide Flow Cytometry Kit (ab139418, Abcam) as previously described^80^. Cells were cultured in 60-mm dishes, after 24 h cells were cultured for an additional 24 h in the absence (control) or presence of the different newly synthesized derivatives (IC_50_ value). The cells were then harvested and fixed in a 100% ice cold ethanol at + 4 °C for at least 2 h. After rewashing with PBS, the cells were incubated with a 200 μl 1× Propidium Iodide (PI) + RNase Staining Solution for 30 min at room temperature in the dark. The DNA content in each cell nucleus was determined by a FACS Calibur flow cytometer (BD Biosciences, Franklin Lakes, NJ, USA). Finally, Cell cycle phase distribution was analyzed using Cell Quest Pro software (BD Biosciences) showing collected propidium iodide fluorescence intensity on FL2.

#### Annexin V-FITC apoptosis assay

Apoptosis assay was performed with an Annexin V-FITC/PI double staining apoptosis detection kit (K101, Biovison) using a flow cytometer^81^. Cells were cultured in 60-mm dishes, after 24 h cells were cultured for an additional 24 h in the absence (control) or presence of the different newly synthesized derivatives (IC_50_ value). Cells were harvested by the trypsinization, washed twice with 4 °C PBS, and re-suspended in the binding buffer. Subsequently, the Annexin V-FITC and Propidium iodide (PI) solutions were added to stain the cells before the analysis by the flow cytometry, where a minimum of 10,000 cells per sample were acquired. The Annexin V-FITC binding (FL1) and PI (FL2) were analyzed, using the Cell Quest Pro software (BD Biosciences).

#### Molecular docking

Jaguar was used for generating all possible tautomeric and stereo-isomeric stats for the structures^82^. Crystal structures of *P*-glycoprotein protein was taken from the protein data bank bonded with 5-florouracil as reference drug. All ligands were imported into Ligprep module and redocked into appropriate binding sites using Glide’s module. The Glide-tool was applied to perform the molecular docking, then a grid for protein charged using the default aspects of force field. The (SP) scoring function produced for study the binding affinity, and then charged with Charm force field. The low-root-square-devotion RMSD score utilized to get the other poses. Schrodinger builder were applied to draw.

#### Statistics

Statistical analysis and figures were performed by GraphPad Prism 5.01 (Graph Pad software, San Diego, CA. USA).

## Conclusions

The newly synthesized benzochromene derivatives (**4a–c, 4g,** and **4h**) showed a potent cytotoxic effect against both non-resistant cancer cells (PC-3, SKOV-3, & HeLa), resistant cancer cells (MCF-7/ADR) and were weakly active against non-cancerogenic cells (HFL-1, WI-38). In addition, compounds **4a–c** showed a potent inhibitory effect of the *P-gp* levels and function in MCF-7/ADR. The Rh123 accumulation assays showed that compounds **4a–c** effectively inhibited *P*-pg production and efflux function. Furthermore, compounds **4a–c** induced arrest of MCF-7/ADR cells at S and G2/M phases inducing apoptosis. To explore the possible binding interactions of the most potent anti-proliferative **4a–c** compounds with *P*-glycoprotein, exhibited greater efficiency of binding compared to reference inhibitor, and engaging key residues of binding pocket similarly to reference inhibitor. The Physicochemical parameters of the active compounds were assessed, indicating favorable values for lipophilicity, solubility, and adherence to rule of toxicity. Furthermore, the Boil-Egg method suggested high gastrointestinal absorption and no permeability to the blood–brain barrier for the tested compounds. Considering their promising activity against *p*-gp and favorable physicochemical properties. These targeted compounds could be used as lead compounds in the development of more potent and pharmacokinetically stable drugs in the future.

### Supplementary Information

Supplementary Figures.

## Data Availability

All data generated or analysed during this study are included in this published article [and its supplementary information files.
