# Supplementary material for: Design, synthesis and bioactivity study on oxygen-heterocyclic-based pyran analogues as effective P-glycoprotein-mediated multidrug resistance in MCF-7/ADR cell
Source: Sci Rep. 2024 Mar 31;14:7589. doi: 10.1038/s41598-024-56197-w (PMC10981727; doi:10.1038/s41598-024-56197-w)
Supplement: Supplementary file 1 — Supplementary Figures. [file 41598_2024_56197_MOESM1_ESM.docx]

**Design, synthesis and bioactivity study on oxygen-heterocyclic-based pyran analogues as effective *P*-glycoprotein-mediated multidrug resistance in MCF-7/ADR cell**

**Ashraf H.F. Abd El-Wahab ^a^, Rita M.A. Borik ^a^, Al-Anood M. Al-Dies,^b^ Ahmed M. Fouda ^c^,**  **Hany M. Mohamed ^a^, R.A. El-Eisawy ^d,e^, Ahmed Mora ^d^, Mohammed A.A. El-Nassag ^d^, Ahmed M. A. I. Abd El-Hady ^d^,Ahmed A. Elhenawy ^d,f,,^*, Ahmed M. El-Agrody ^d,^***

*^a^ Chemistry Department, Faculty of Science, Jazan University, B.O. Box 2097, Jazan 45142, Kingdom of Saudi Arabia*

*^b^ Chemistry Department, Umm Al-Qura University, Al-Qunfudah University College, Al-Qunfudah 21912, Saudi Arabia*

*^c^ Chemistry Department, Faculty of Science, King Khalid University, Abha 61413, Saudi Arabia*

***^d^*** *Chemistry Department, Faculty of Science, Al-Azhar University, Nasr City 11884, Cairo, Egypt*

*^e^ Chemistry Department, Faculty of Science and Art, Al-Baha University, Al-Baha, 1988, Saudi Arabia*

*^f^ Chemistry Department, Faculty of Science and Art, AlBaha University, Al Bahah 65731, Saudi Arabia*

^⁎^ Corresponding author: Ahmed M. El-Agrody (elagrody_am@azhar.edu.eg), Ahmed A. Elhenawy ([elhenawy_sci@hotmail.com](mailto:elhenawy_sci@hotmail.com))

| **Table of Contents:** | **Page** |
| --- | --- |
| **Figure S1:**  ^1^H NMR of cpd. **(4c)***.* | S3 |
| **Figure S2:** ^13^C NMR of cpd. **(4c)**. | S4 |
| **Figure S3:** ^13^C NMR/APT of cpd. **(4c)**. | S5 |
| **Figure S4:**  ^1^H NMR of cpd. **(4f)***.* | S6 |
| **Figure S5:** ^13^C NMR of cpd. **(4f)**. | S7 |
| **Figure S6:** ^13^C NMR/APT of cpd. **(4f)**. | S8 |
| **Figure S7:**  ^1^H NMR 8.5-6.5 ppm of cpd. **(4h)***.* | S9 |
| **Figure S8:** ^1^H NMR of cpd. **(4h)***.* | S10 |
| **Figure S9:** ^13^C NMR of cpd. **(4h)**. | S11 |
| **Figure S10:** ^1^H NMR 8.5-6.5 ppm of cpd. **(4i)***.* | S12 |
| **Figure S11:** ^1^H NMR of cpd. **(4i)***.* | S13 |
| **Figure S12:** ^13^C NMR of cpd. **(4i)***.* | S14 |
| **Figure S13:** ^1^H NMR 8.5-6.5 ppm of cpd. **(4j)***.* | S15 |
| **Figure S14:** ^1^H NMR of cpd. **(4j)***.* | S16 |
| **Figure S15:** ^13^C NMR of cpd. of cpd. **(4j)***.* | S17 |
| **Figure S16:** ^1^H NMR 8.5-6.5 ppm of cpd. **(4k)***.* | S18 |
| **Figure S17:** ^1^H NMR of cpd. **(4k)***.* | S19 |
| **Figure S18:** ^13^C NMR of cpd. **(4k)***.* | S20 |
| **Figure S19:** ^1^H NMR 8.5-6.5 ppm of cpd. **(4l)***.* | S21 |
| **Figure S20:** ^1^H NMR of cpd. **(4l)***.* | S22 |
| **Figure S21:** ^13^C NMR of cpd. **(4l)***.* | S23 |


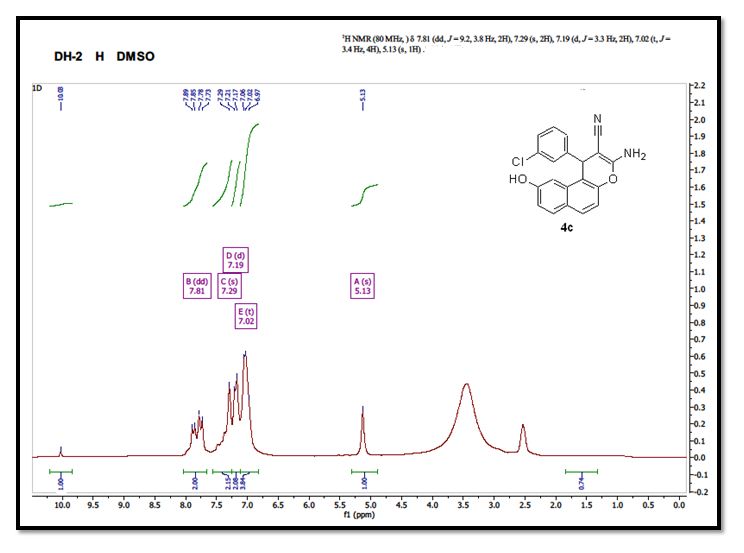


**Figure S1:** ^1^H NMR of cpd. **(4c)***.*


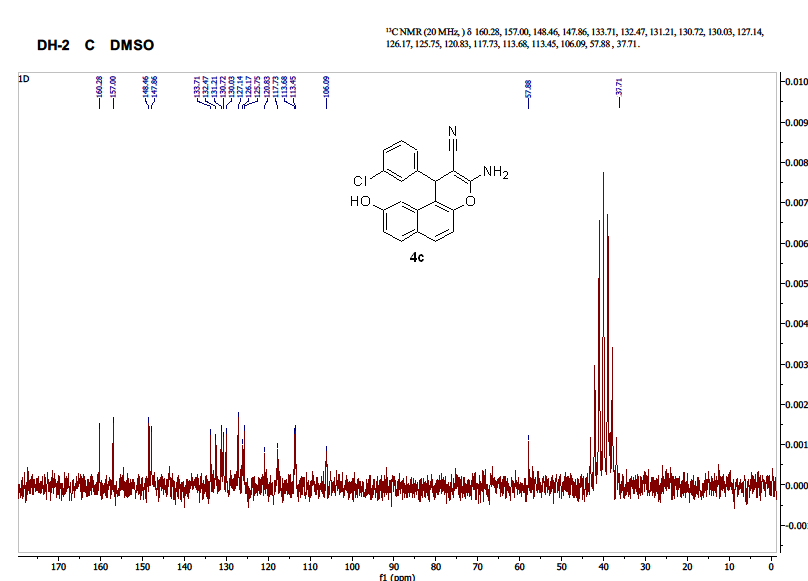


**Figure S2:** ^13^C NMR of cpd. **(4c)**.


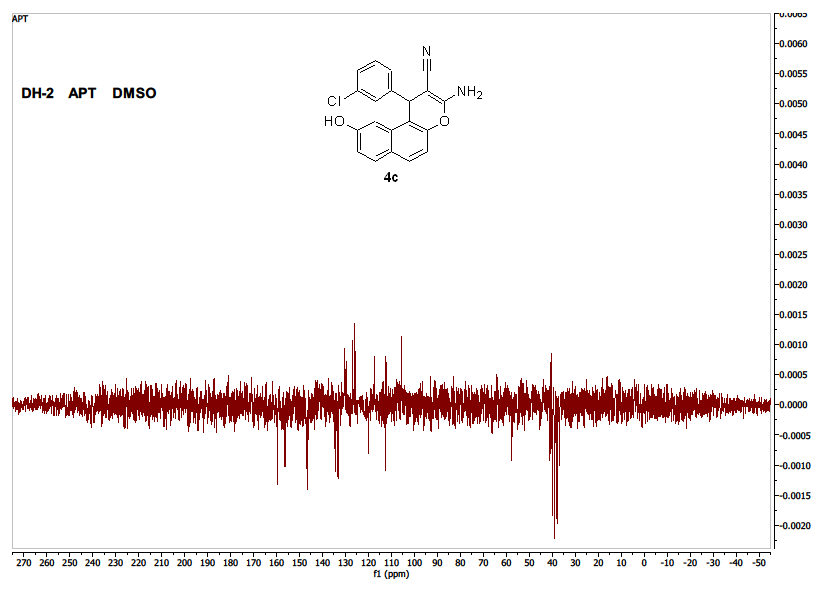


**Figure S3:** ^13^C NMR/APT of cpd. **(4c)**.


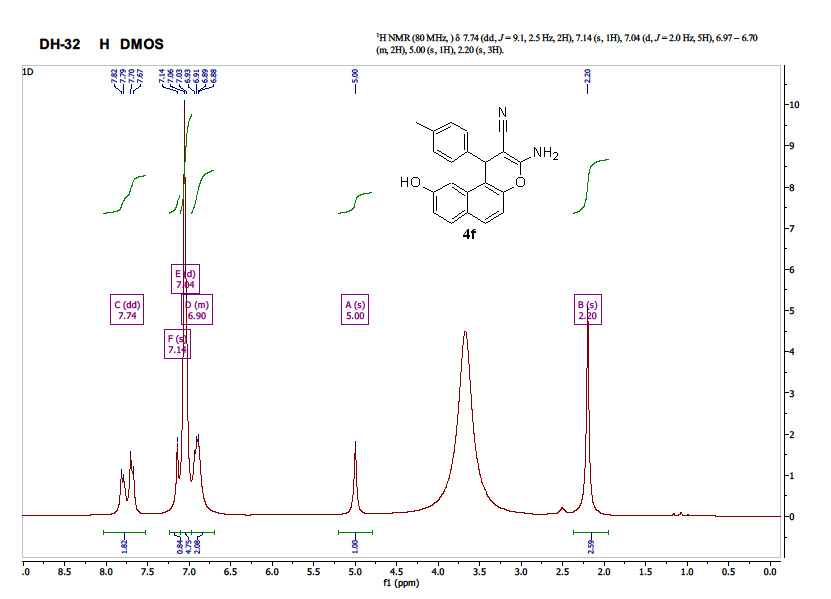


**Figure S4:**  ^1^H NMR of cpd. **(4f)***.*


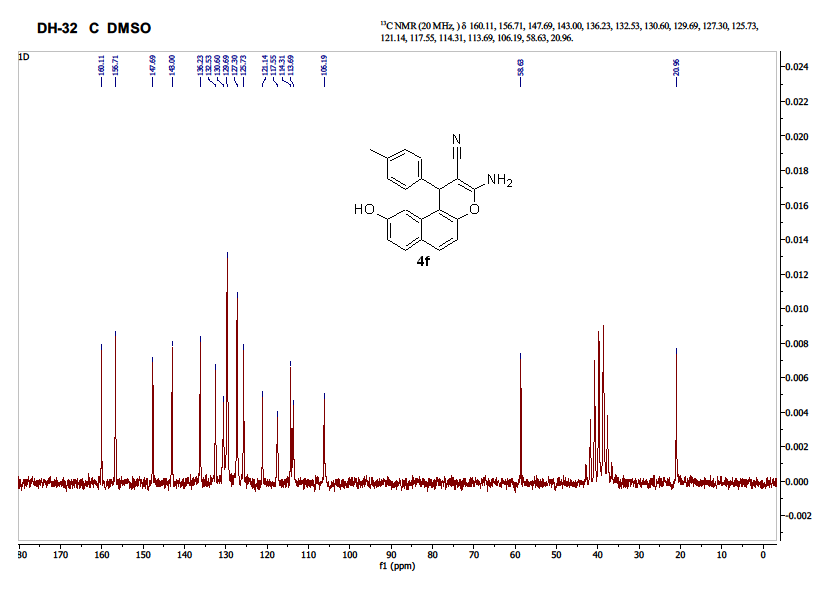


**Figure S5:**  ^13^C NMR of cpd. **(4f)***.*


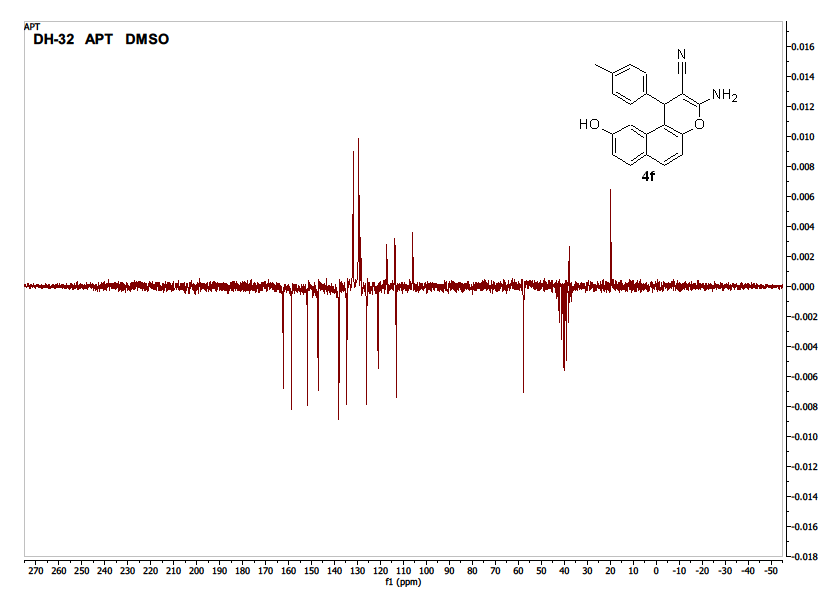


**Figure S6:**  ^13^C NMR/APT of cpd. **(4f)***.*


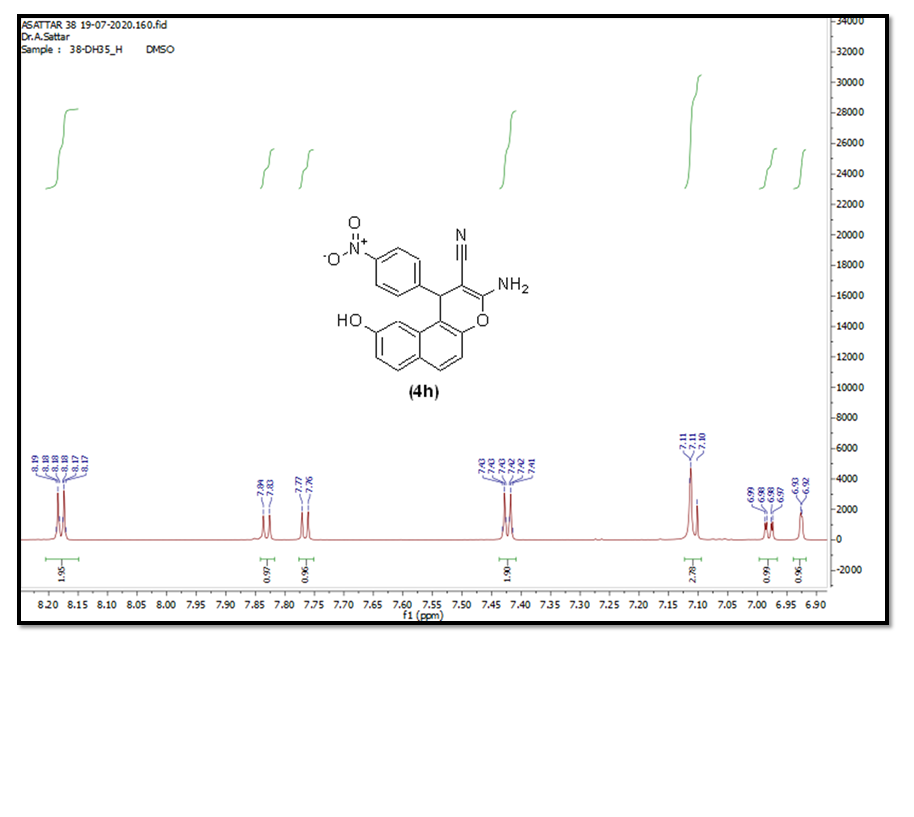


**Figure S7:** ^1^H NMR 8.5-6.5 ppm of cpd. **(4h)***.*


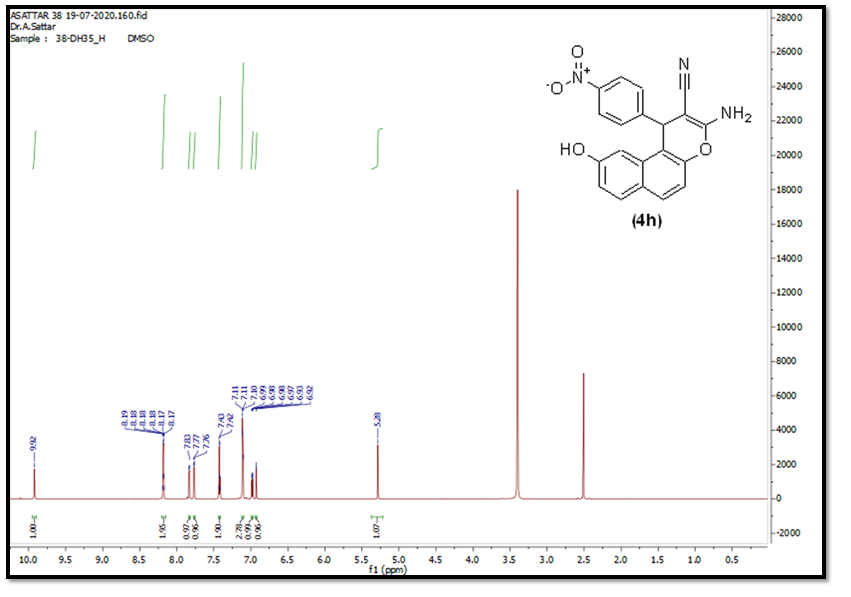


**Figure S8:** ^1^H NMR of cpd. **(4h)*.***


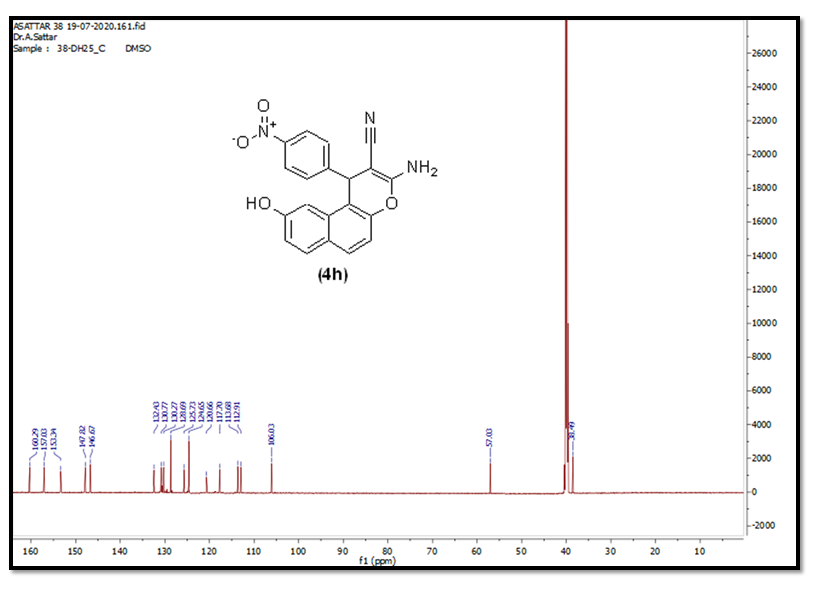


**Figure S9:** ^13^C NMR of cpd. **(4h)**.


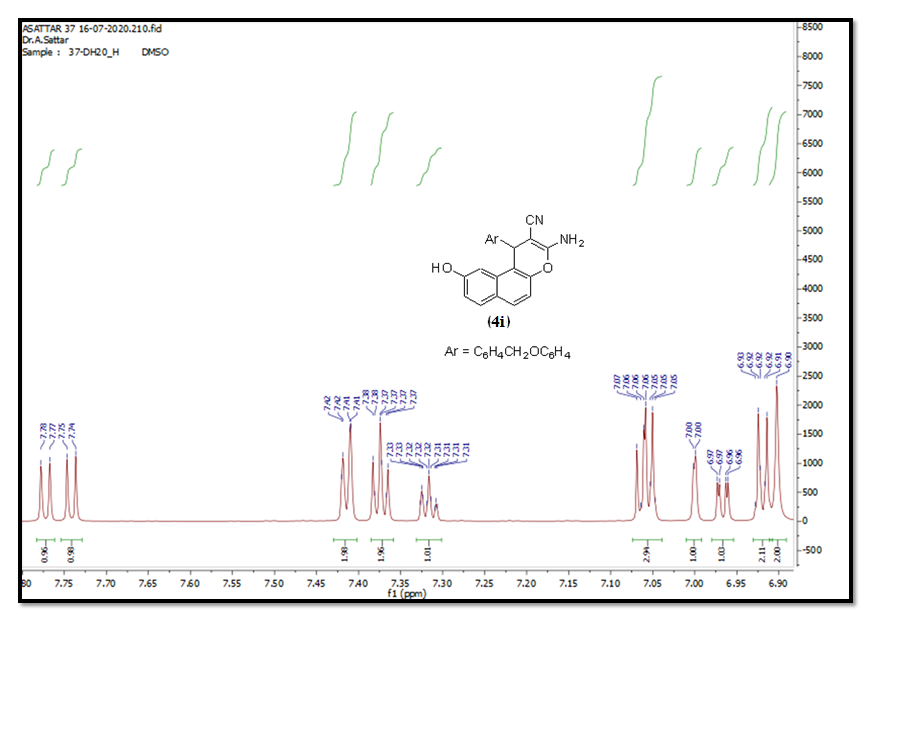


**Figure S10:** ^1^H NMR 8.5-6.5 ppm of cpd. **(4i)***.*


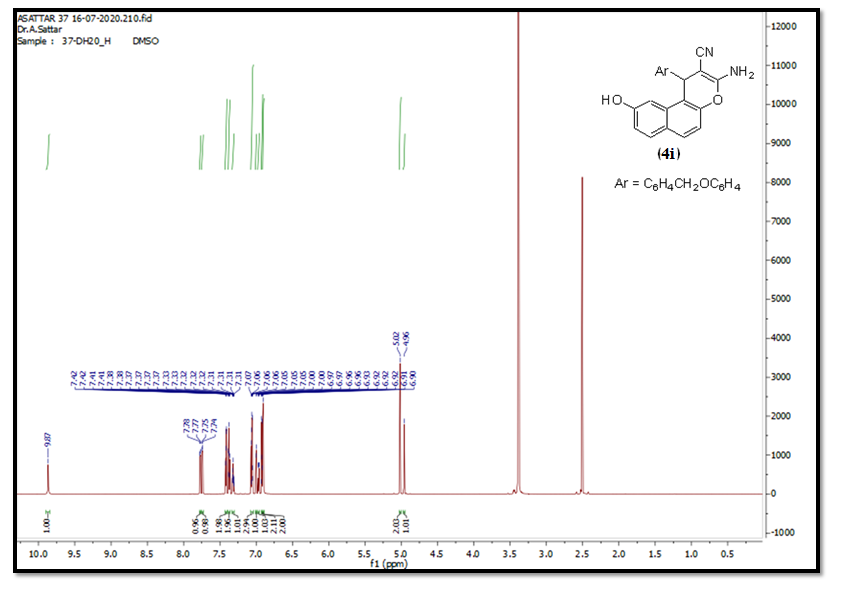


**Figure S11:** ^1^H NMR of cpd. **(4i)***.*


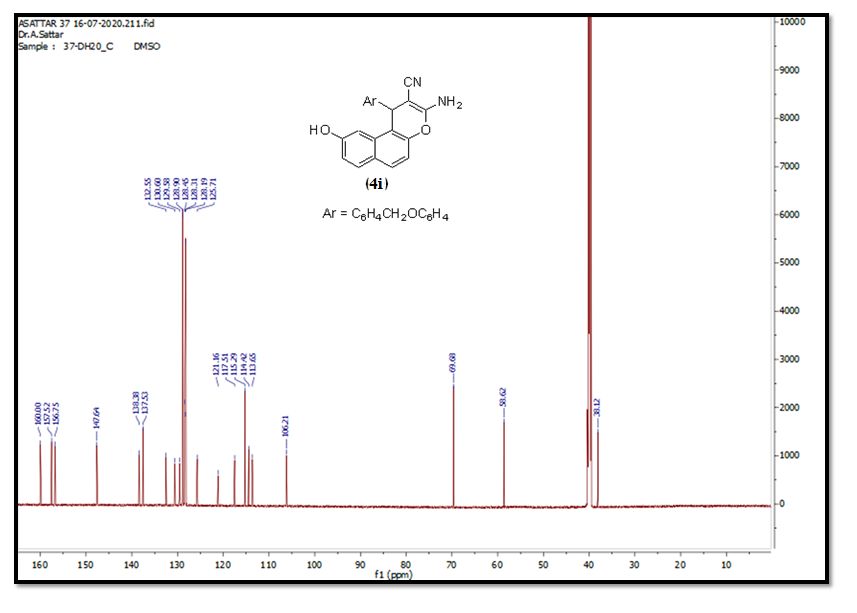


**Figure S12:** ^13^C NMR of cpd. **(4i)***.*


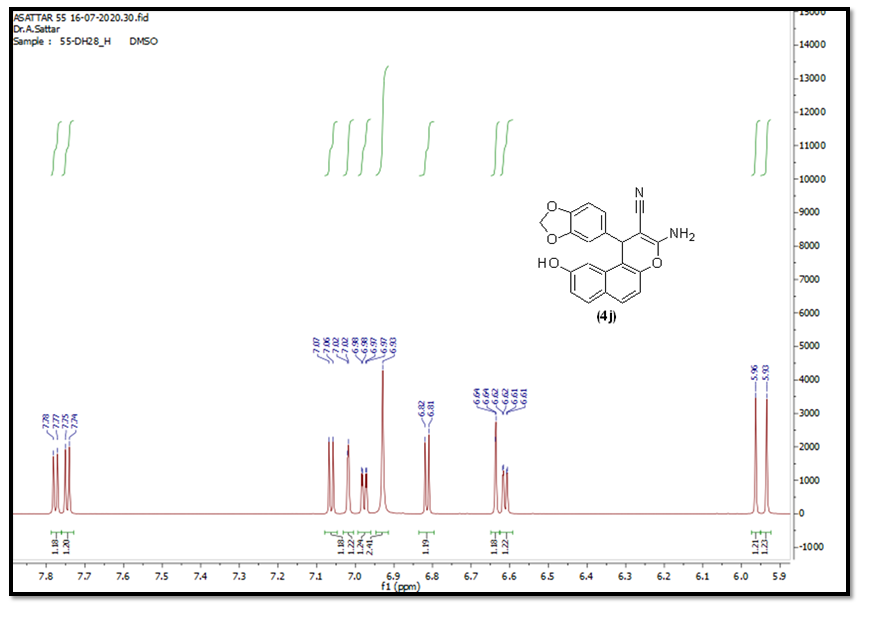


**Figure S13:** ^1^H NMR 8.5-6.5 ppm of cpd. **(4j)***.*


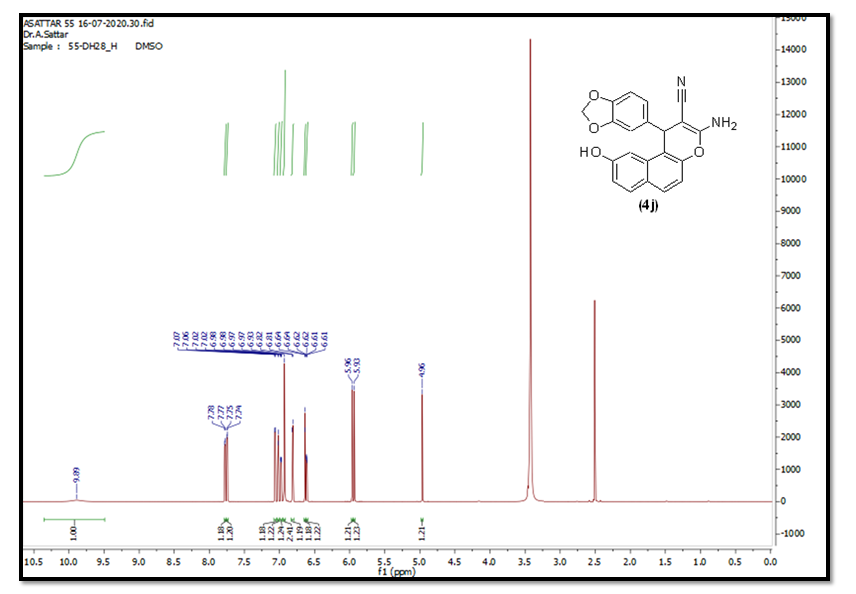


**Figure S14:** ^1^H NMR of cpd. **(4j)***.*


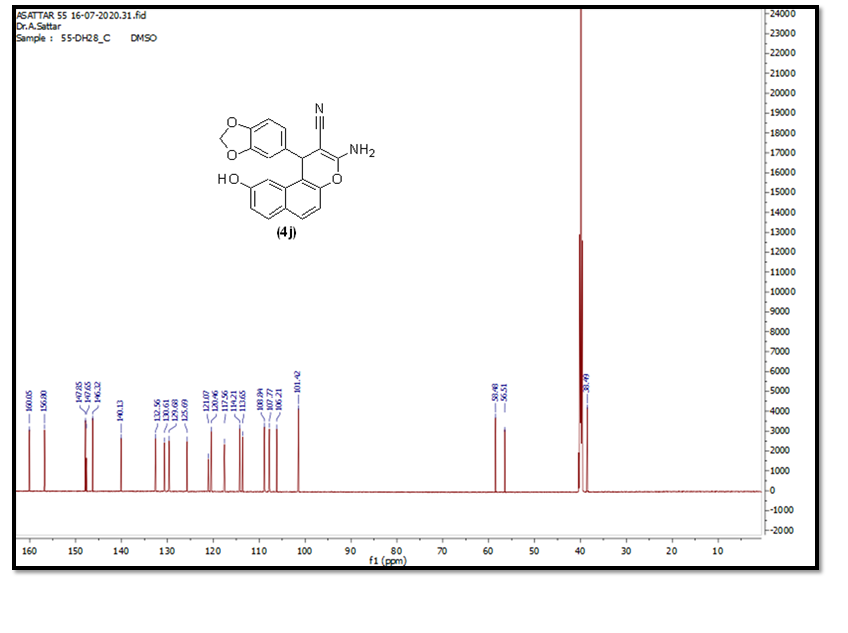


**Figure S15:** ^13^C NMR of cpd. **(4j)***.*


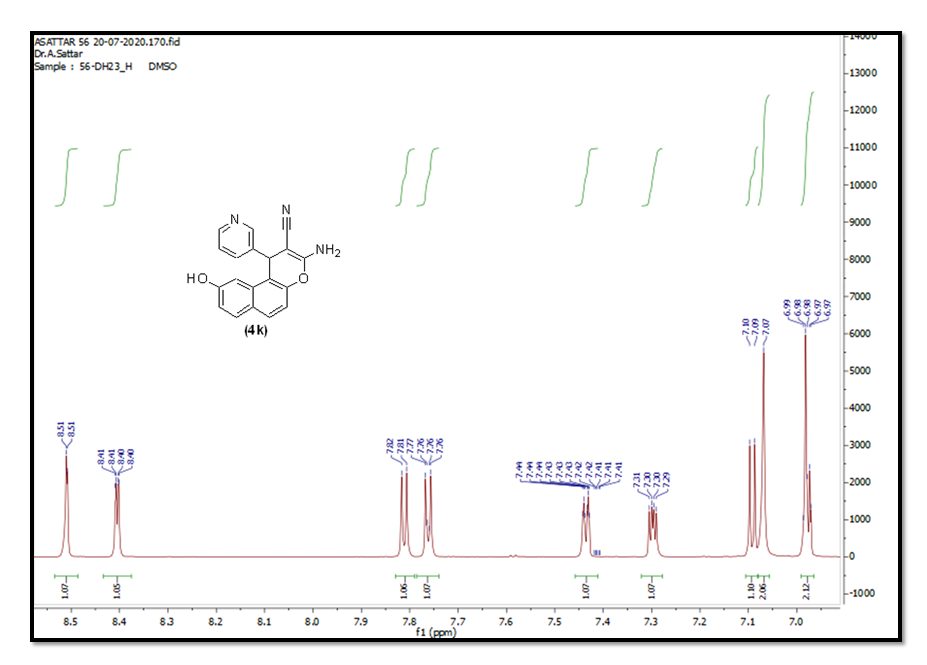


**Figure S16:** ^1^H NMR 8.5-6.5 ppm **(4k)***.*


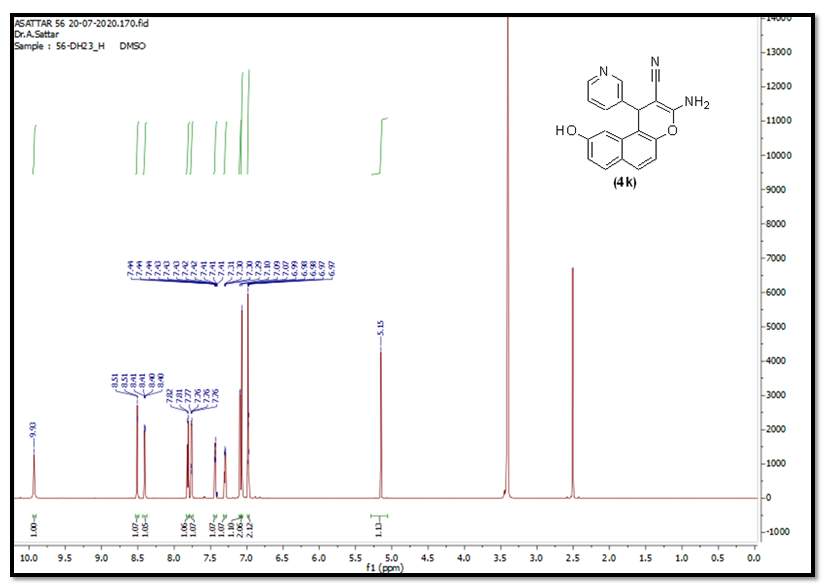


**Figure S17:** ^1^H NMR of cpd. **(4k)***.*


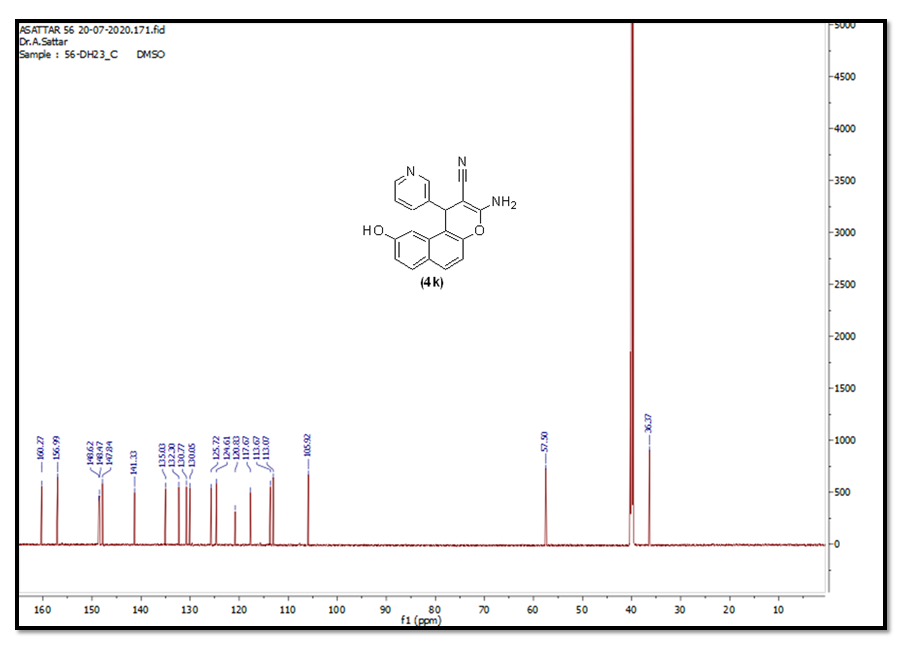


**Figure S18:** ^13^C NMR of cpd. **(4k)***.*

**
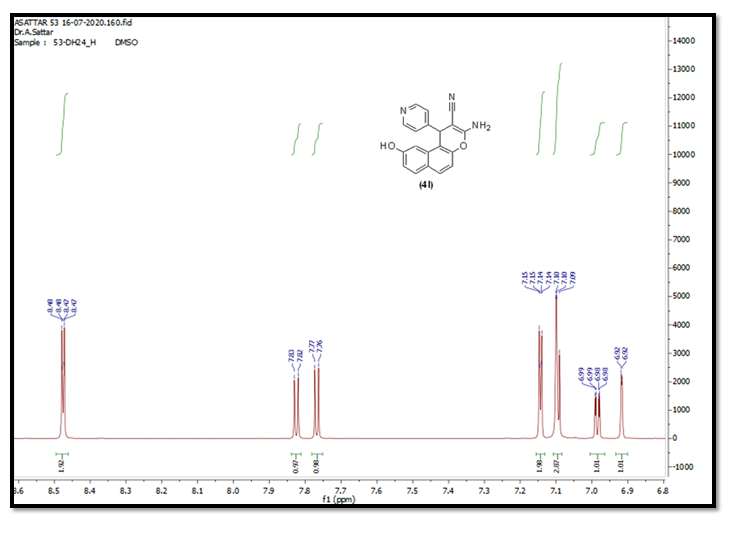
**

**Figure S19:** ^1^H NMR 8.5-6.5 ppm of cpd **(4l)***.*


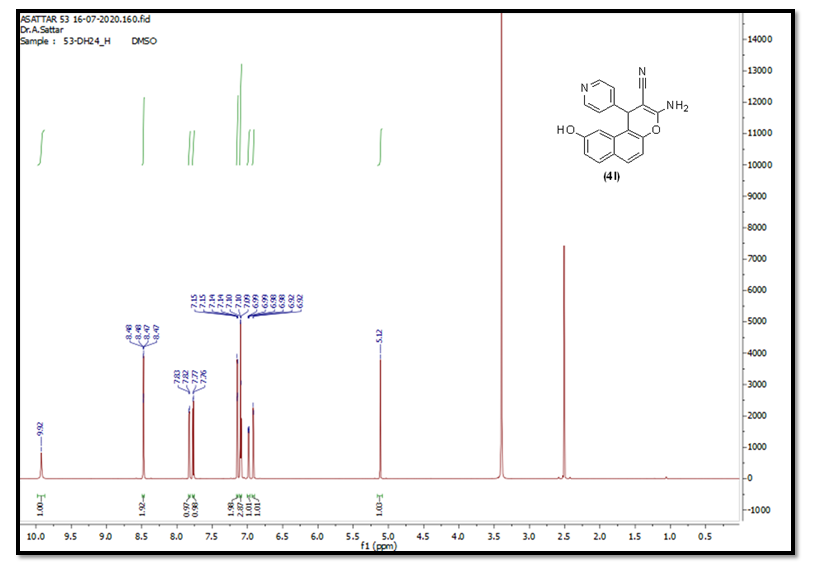


**Figure S20:** ^1^H NMR of cpd **(4l)***.*


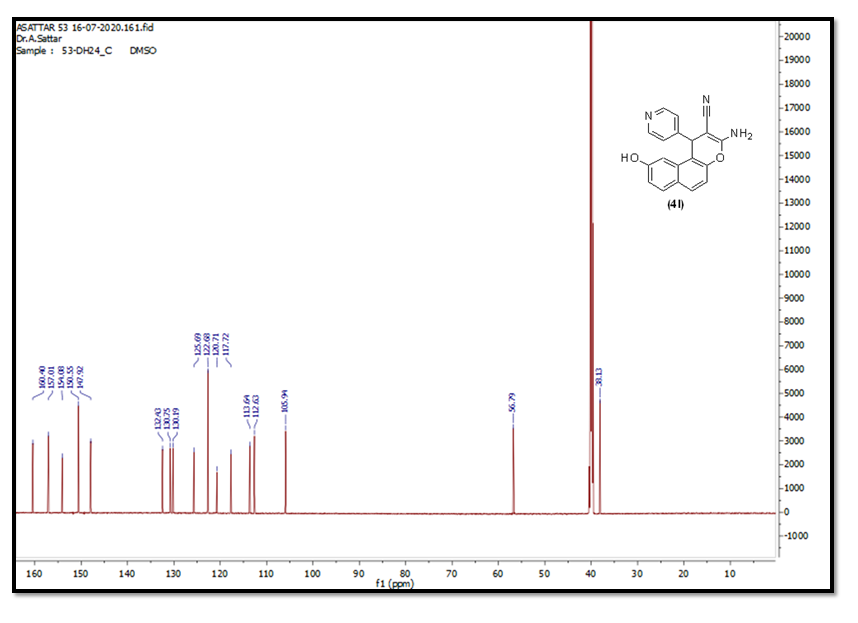


**Figure S21:** ^13^C NMR of cpd. **(4l)***.*
